# Country‐wide genetic monitoring over 21 years reveals lag in genetic recovery despite spatial connectivity in an expanding carnivore (Eurasian otter, *Lutra lutra*) population

**DOI:** 10.1111/eva.13505

**Published:** 2022-11-15

**Authors:** Nia E. Thomas, Frank Hailer, Michael W. Bruford, Elizabeth A. Chadwick

**Affiliations:** ^1^ Organisms and Environment Research Division, School of Biosciences Cardiff University Cardiff Wales UK

**Keywords:** gene flow, genetic monitoring, microsatellites, population genetics, population recovery, time lag

## Abstract

Numerous terrestrial mammal species have experienced extensive population declines during past centuries, due largely to anthropogenic pressures. For some species, including the Eurasian otter (*Lutra lutra*), environmental and legal protection has more recently led to population growth and recolonization of parts of their historic ranges. While heralded as conservation success, only few such recoveries have been examined from a genetic perspective, i.e. whether genetic variability and connectivity have been restored. We here use large‐scale and long‐term genetic monitoring data from UK otters, whose population underwent a well‐documented population decline between the 1950s and 1970s, to explore the dynamics of a population re‐expansion over a 21‐year period. We genotyped otters from across Wales and England at five time points between 1994 and 2014 using 15 microsatellite loci. We used this combination of long‐term temporal and large‐scale spatial sampling to evaluate 3 hypotheses relating to genetic recovery that (i) gene flow between subpopulations would increase over time, (ii) genetic diversity of previously isolated populations would increase and that (iii) genetic structuring would weaken over time. Although we found an increase in inter‐regional gene flow and admixture levels among subpopulations, there was no significant temporal change in either heterozygosity or allelic richness. Genetic structuring among the main subpopulations hence remained strong and showed a clear historical continuity. These findings highlight an underappreciated aspect of population recovery of endangered species: that genetic recovery may often lag behind the processes of spatial and demographic recovery. In other words, the restoration of the physical connectivity of populations does not necessarily lead to genetic connectivity. Our findings emphasize the need for genetic data as an integral part of conservation monitoring, to enable the potential vulnerability of populations to be evaluated.

## 1 INTRODUCTION

Many large mammal species have experienced population declines during past centuries, largely due to anthropogenic causes (Cardillo et al., 2005). Some of these declines are now being reversed due to protective legislation, and population recoveries are being observed (Chapron et al., 2014). These declines and subsequent re‐expansions into historically occupied ranges provide natural experiments to measure how genetic diversity and structure change both temporally and spatially during population growth, allowing for the testing of theoretical predictions from simulation studies (Hagen et al., 2015).

Theoretical studies have shown that population expansions are likely to be accompanied by changes in genetic diversity (Excoffier & Ray, 2008), which may differ from the changes caused by demographic growth alone (Excoffier et al., 2009). During population expansions, sequential founder events can cause unusual phenomena, including reduced genetic diversity at the wavefront due to random genetic drift, combined with the relative isolation of the founder individuals from the rest of the population (Excoffier & Ray, 2008). As populations continue to expand, connectivity may be established between previously isolated demes. If this spatial connectivity results in effective gene flow, reductions in spatial population structuring between differentiated groups should be observed, resulting in increased genetic diversity within groups (Ibrahim et al., 1996; Excoffier et al., 2009). Different population genetic metrics will respond with different speeds to such re‐establishment of gene flow. A leading, i.e. early indicator of such genetic recovery is allelic richness, while heterozygosity‐based statistics will respond more slowly and thus are lagging indicators (Nei, 1987).

Wildlife populations that have experienced large declines are often fragmented in the process, leading to differentiation through genetic drift (Manel et al., 2004; Rueness et al., 2003). Subsequent recovery of these populations through expansion is therefore predicted to reduce spatial genetic structure through gene flow by re‐establishing connectivity between previously isolated genetically differentiated units (Hagen et al., 2015). However, detecting this connectivity requires temporal sampling of the population to determine changes in gene flow, genetic differentiation and genetic diversity between and within subpopulations over time. Such information quantifying the dynamics of range expansions is vital in order to make predictions about the nature of population recoveries, and to develop and monitor progress towards realistic management goals.

The Eurasian otter (*Lutra lutra*; henceforth referred to as otter) is a largely piscivorous mesocarnivore, which feeds both in freshwater and coastal habitat (Kruuk, 1995). Otters in the United Kingdom are undergoing a population expansion, recovering from a well‐documented large‐scale decline that occurred in the second half of the 20th century (Crawford, 2010; Strachan, 2015). This decline was largely attributed to the use and bioaccumulation of pesticides and other industrial compounds such as dieldrin and polychlorinated biphenyls (PCBs), although habitat loss and direct persecution through hunting may also have contributed (Conroy & Chanin, 2000; Mason & Macdonald, 2004). Resulting population declines since the 1950s led to the extinction of otters across large areas of England (Figure S1), and previous genetic analyses have shown strong north–south genetic differentiation, as well as several genetically distinct subpopulations (Hobbs et al., 2011; Stanton et al., 2014). This structuring likely reflects both remnant populations, which survived the decline (strongholds in South West England, Wales and Scotland), and past reinforcement programmes (in northeast and southern England; Hobbs et al., 2011).

A wide range of threats pose significant, new and persistent challenges to freshwater biodiversity (Reid et al., 2018), including both direct and indirect threats to otters (O'Rourke et al., 2022). Despite these challenges, national otter survey data (Mathews et al., 2018) indicates that the UK otter population has been expanding both in range and population size since the 1980s (Figure S1). This population recovery is attributed to legislative protection for both otters (Wildlife and Countryside Act, 1981) and their freshwater habitats (EC Habitats Directive, 1992), and reduction in environmental pollution (Kean et al., 2021). The spatial and demographic recovery evidenced by national otter survey data has resulted in an apparently spatially contiguous population in the UK, with previously isolated subpopulations now re‐joined (Mathews et al., 2018). It remains unclear, however, to what degree this apparent spatial contiguity might have translated into genetically connected populations. Using samples and data collected over a twenty‐one‐year period (1993–2014) during population recovery, we focus on the otter as an ideal case study with which to test the following hypotheses:

1. Genetic structuring across the study area will weaken over time as demographic population recovery and spatial expansion proceed, reconnecting previously isolated subpopulations.
2. Gene flow between subpopulations/regions will increase over time with increased contact due to range expansions.
3. Genetic diversity of previously isolated subpopulations will increase, as gene flow between neighbouring subpopulations allows the influx of new alleles and changes allele frequencies of standing genetic variation within populations.

## 2 METHODS

### 2.1 Sample collection and selection

Road‐killed otter carcasses from across Wales and England were collected and their locations recorded as part of the national Cardiff University Otter Project programme (www.cardiff.ac.uk/otter‐project). Muscle samples were taken from the hind leg and stored in ethanol at −20°C.

Samples and associated metadata were selected to represent five time points between 1993 and 2014, separated by approximately 5‐year intervals (1993–5, 1998–2000, 2004, 2009 and 2014). This maximized the overall temporal span (within the constraint of samples available to the Cardiff University Otter Project at the time of analysis) while providing a sufficient number of discrete time points to facilitate the interpretation of trends. A minimum of 3 years separation between time points ensured appropriate temporal independence, based on a typically assumed otter generation time of 3 years (Randi et al., 2003; although more conservative models suggest 7.6 years; calculated according to Pacifici et al., 2013). Earlier temporal subsets were pooled across three consecutive years due to insufficient sample availability within individual years. For time points up to 2004, genotype data were used from a previous study (Hobbs et al., 2011), including 1993–5 (*n* = 28), 1998–2000 (*n* = 100) and 2004 (*n* = 79). For 2009, 97 samples were genotyped and 7 existing genotypes were included from Stanton et al., 2014; from 2014, 96 samples were genotyped (see Table S2 for data allocation to each study).

When selecting the additional samples to genotype for this study, to avoid spatial pseudoreplication, which might arise through truly random sampling, we randomly selected one sample from every 20 km grid square in which at least one dead otter had been collected that year. We chose a 20 km grid based on otter range size, for which estimates vary between 7.5 km ± 1.5 km (Néill et al., 2009) and 20–30 km (Erlinge, 1968). The resulting dataset comprised 407 individuals, including 236 males, 168 females and three individuals where neither sex nor age class could be determined (males: 147 adults, 78 subadults and 10 juveniles along with one individual of unknown age class; females: 91 adults, 64 subadults and 10 juveniles, along with three individuals of unknown age class). Due to our use of (primarily) roadkill as a sampling mechanism, it is possible that our dataset is biased toward younger male otters, relative to the wild population (Philcox et al., 1999), although without any means of independent verification of population demography, this is not possible to ascertain.

**FIGURE 1:**
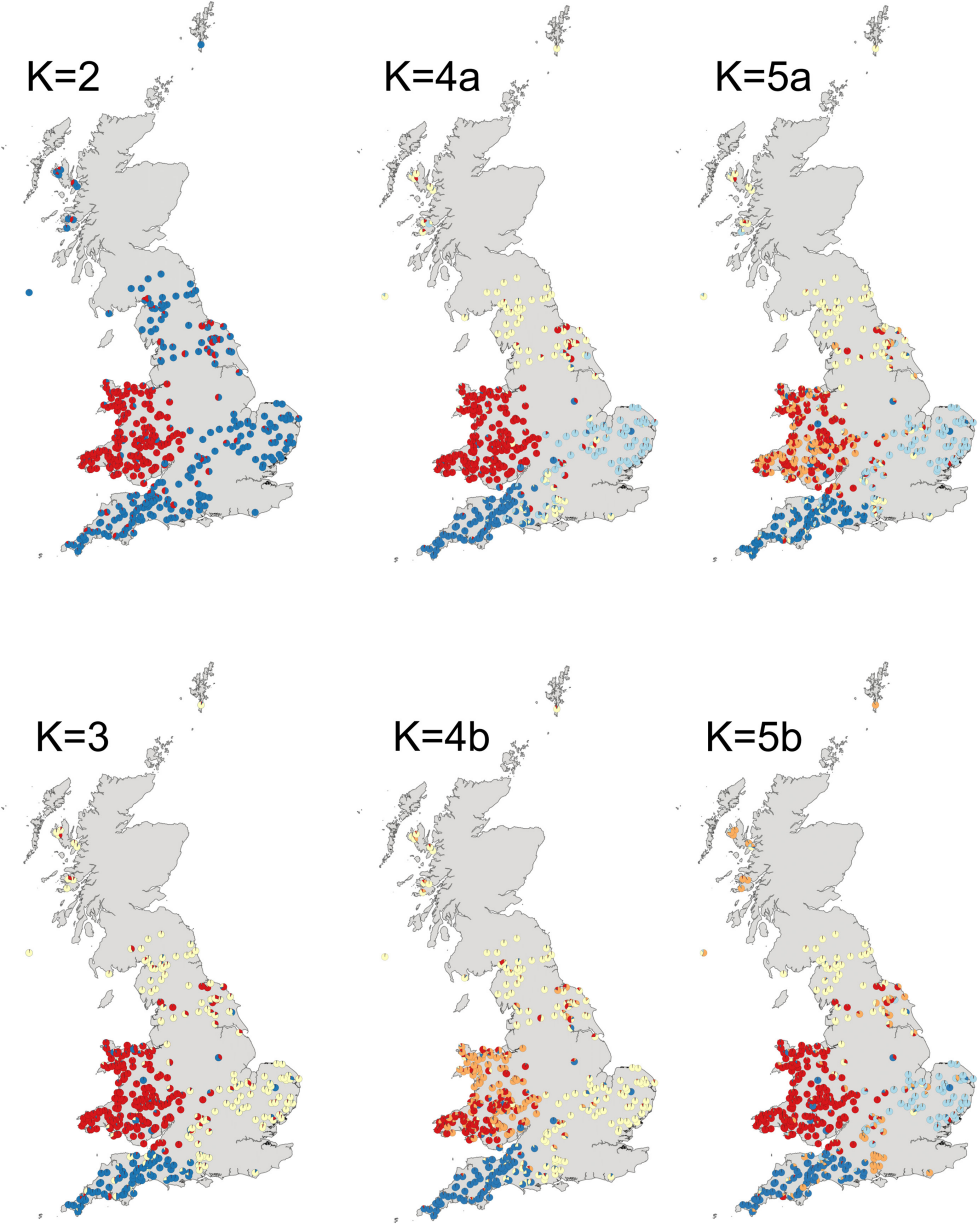
Genetic clusters (K2–5) identified in UK otters sampled across 1999–2014 using a Bayesian approach in structure. Circles show the location of each otter in the dataset and the colours indicate the proportion of each genetic cluster to each individual belongs to. For K = 4 and K = 5, a denotes the major mode and b the minor mode where not all 10 repeat runs agreed, see Figure S4 for the number of runs attributed to each mode by CLUMPP.

Each sample was mapped as a point location in ESRI ArcGIS 10.3 (Figure 1), and each point allocated to a River Basin District (as specified in the Water Framework Directive Cycle 2, Environment Agency, 2015; Natural Resources Wales, 2015, which are based on groupings of river catchments). Otters typically occupy linear home ranges along freshwater habitats such as rivers (Kruuk, 1995), hence we chose watershed‐based spatial aggregations of data as likely to provide an ecologically relevant unit. Aggregation of data within smaller (e.g. catchment‐based) units provided insufficient sample size for some of our analyses. RBDs provided a suitable sample size in most areas, although aggregation of adjacent RBDs was required in some areas to ensure adequate sample size for certain analyses (Table 1). We defined these spatial units as ‘RBD regions’ and combined these with the temporal data (sampling year) to yield 23 spatial–temporal groupings (STGs) for further analysis.

**TABLE 1:** Geographic regions used in genetic diversity and differentiation analysis of UK Eurasian otters (*Lutra lutra*).

RBD region	Included river basin districts (RBDs)	*N*	Land area (km^2^)
Eastern	Anglian, South East, Thames	74	50,226
Northern	Humber, North West, Northumbria, Solway Tweed	59	61,601
Severn	Severn	84	21,056
South West	South West	77	18,191
Western Wales	Dee and Western Wales	102	14,715
Other	Ireland and Scotland	11	NA

*Note*: RBD: Regions are based on amalgamations of River Basin Districts (RBDs) as defined in the Water Framework Directive Cycle 2 (Environment Agency, 2015; Natural Resources Wales, 2015), *N* is the number of otters genotyped and Land Area is the total km^2^ of land within an RBD region (no Land Area is provided for the ‘other’ RBD region as it covers a vast and unconnected area of land with very few samples).

### 2.2 DNA extraction and microsatellite genotyping

DNA was extracted from muscle tissue using the Qiagen DNeasy Blood and Tissue Kit (Qiagen) following the manufacturer's guidelines. Samples were genotyped at 15 microsatellite loci using three multiplexes as in Hobbs et al. (2006). The 15 loci were: Lut 435, 457, 604, 615, 701, 715, 717, 733, 782, 818, 832, 833 and 902 (Dallas et al., 1999; Dallas & Piertney, 1998), and 040T05 and 040T22 (Huang et al., 2005). Polymerase chain reactions were conducted in 10 μl reactions with 1× (5 μl) QIAGEN Multiplex PCR Master Mix (Qiagen), 0.2 μM of each primer, sterile water and 2 μl of template DNA. The cycling conditions used were as follows: 95°C for 15 min; 29 cycles of 94°C for 30 s, 58°C for 90 s, 72°C for 60 s; and a final extension at 60°C for 30 min. Samples selected from the previous studies (Hobbs et al., 2011; Stanton et al., 2014) had been genotyped at the same 15 loci using the same conditions. Fluorescently labelled PCR products were sent to DNA Sequencing Services (Dundee, Scotland) for fragment analysis using an Applied Biosystems 3730xl DNA analyser and visualized using Genemapper 4.0 (Applied Biosystems, 2006). A subset of 14 previously genotyped samples (Hobbs et al., 2011) was also re‐analysed to allow calibration of fragment size scoring between the three studies: Stanton et al. (2014) calibrated their data to Hobbs et al. (2011), therefore calibration using the 14 samples from Hobbs et al. (2011) was sufficient to bring all three sets of data together. Despite the known challenges of combining microsatellite datasets (Ellis et al., 2011), this procedure enabled adjustment of allele sizes to account for variation between sequencing platforms and scorers, allowing the dataset to be analysed as a whole (see Section 3 for details).

### 2.3 Genetic variability by locus, within spatiotemporal groupings (STGs) of River Basin District (RBD) regions

Genotyping errors and null allele frequencies were estimated using Microchecker V2.2.3 (Van Oosterhout et al., 2004). Each spatial–temporal grouping (STG) was run independently, and loci were only removed if they were identified as having null alleles in the majority of regions at a specific time point (D'Urban Jackson et al., 2017). Null alleles are a potential source of bias during the estimation of population differentiation (F_ST_) therefore it is important to identify them if present. An exact test of Hardy–Weinberg equilibrium (HWE) and a test for linkage disequilibrium (LD) between all pairs of loci were conducted in ARLEQUIN 3.5 (Excoffier & Lischer, 2010). Deviation from HWE was estimated using 1,000,000 Markov chain steps and 100,000 dememorisation steps. Linkage disequilibrium between loci was estimated using 10,000 permutations with the number of random initial conditions set to 2 and a significance level of 0.05. These analyses were run on the whole dataset (i.e. for all STGs combined) initially and then repeated on the samples in each STG individually, for LD only time points 2009 and 2014 were included in the analysis as a representative sample as for true LD one would expect the same result at any given time point and these data had full spatial coverage over the study area and the largest sample size.

Genetic diversity per locus was estimated using MICROSATELLITE TOOLKIT (Park, 2001). Unbiased expected heterozygosity (Nei, 1987), observed heterozygosity and number of alleles per locus were calculated along with the mean of each of these statistics. A paired 2‐sample *t*‐test was used to test for a significant difference between the expected and observed heterozygosity using R studio (R version 3.4.3, R Core Team, 2017).

Genetic diversity was estimated for each STG using multiple diversity statistics. We used MICROSATELLITE TOOLKIT (Park, 2001) to estimate an unbiased estimator of expected heterozygosity, based on Nei's unbiased gene diversity (Nei, 1987) and observed heterozygosity including standard deviations. Expected heterozygosity and observed heterozygosity are more robust to sample size changes, when the sample size is greater than 5–10 individuals (a threshold that was met in the majority, 21/23 for *N* > 5 and 19/23 for *N* > 10 of our STGs); however, it is particularly important that standard deviations are reported and considered alongside estimates for smaller sample sizes (Pruett & Winker, 2008). HP‐RARE v1.1 (Kalinowski, 2005) was used to estimate allelic richness (A_r_) and private allelic richness (pA_r_) for each STG; initially, this was calculated using the smallest sample size at each individual time point, respectively. As sample size fluctuated both between regions and time points, we considered the bias that these fluctuations might introduce into each analysis. For analyses that were sensitive to such data fluctuations (e.g. allelic richness), we used the resampling approach implemented in HP‐RARE where the resample size was set to the smallest sample size in any RBD region for that year (Pruett & Winker, 2008) to allow comparisons to be made across all RBD regions at a particular time point. A second analysis was run where each dataset was resampled at the smallest sample size across all time points (*N* = 6) to account for sample size bias in the estimations and allow comparisons to be made across all STGs. Additional statistical analyses of genetic diversity estimates were carried out using R; correlations between both allelic richness and private allelic richness under both resampling regimes were assessed using Kendall's Tau (nonparametric rank correlation method that allows for tied values), as was the relationship between observed heterozygosity and RBD region land area.

The inbreeding coefficient (F_IS_) was initially estimated based on all individuals from each STG, using FSTAT version 2.9.3.2 (Goudet, 1995), and tested for significant deviation from 0 using a 1‐sample *T*‐test in R with correction for false discovery rate (FDR) using the Benjamini and Hochberg (1995) method to account for multiple testing. Population structure and admixture can affect F_IS_ estimates, however. For example, if individuals from more than one genetic cluster are analysed as a single population, a deficiency of heterozygotes is likely to be observed due to violation of the Hardy–Weinberg equilibrium (HWE) assumption of a single, randomly mating population (Hardy, 1908; Waples, 2015; Weinberg, 1908). This deficiency produces a positive F_IS_ value and thus a false indication of inbreeding, and is termed the ‘Wahlund effect’ (Wahlund, 1928). In order to distinguish between the Wahlund effect and actual, recent population‐level inbreeding, we therefore recalculated F_IS_ using only individuals identified as belonging to the dominant genetic cluster for the STG, defined as follows. First, cluster assignments and admixture proportions were calculated for each STG using STRUCTURE 2.3.4 (Pritchard et al., 2000), at the smallest value of K, which maximized global likelihood (Kalinowski, 2011). For each STG, we then identified individuals as either belonging to the dominant cluster or to one of the two minor clusters, based on their proportional allocation to each. Individuals not belonging to the major cluster were excluded from the recalculated value of F_IS_, and a comparison of the two F_IS_ estimates allowed us to highlight any results potentially explained by Wahlund effects. Individuals were considered ‘admixed’ if their assignment proportion was less than 0.8 to a single cluster (Heppenheimer et al., 2018; Rutledge et al., 2010), remaining individuals were considered ‘nonadmixed’.

To statistically explore variation in genetic diversity we tested for differences between regions and years in several measures: individual H_o_, A_r_ (calculated within each STG, and resampled at *N* = 6, see above) and individual admixture proportions (using the largest assignment at K = 3 as our indicator), with each measure in turn assigned as the dependent variable. To ensure that our models were not confounded by uneven sampling across region/year groups, we used a GLM approach including both RBD region and year as independent terms and excluded 1994 due to data deficiency. For our models of individual H_o_ and individual admixture proportions, we also included Sex as a categorical variable, to test for sex bias. In preliminary testing for H_o_ and admixture, we incorporated the year:RBD region interaction to evaluate whether putative change over time differed between regions, but in all cases, the interaction was nonsignificant (*p* > 0.05) and in order to simplify model reporting this term was removed from all starting models. For all GLMs, model fit was evaluated by exploration of residuals to check assumptions of normality, homogeneity of variance and absence of leverage. Tukey tests were applied post hoc to test pairwise differences. For models of H_o_ and A_r_, a Gaussian model with an identity link met all model assumptions. Models of admixture failed to meet necessary assumptions and no suitable error family/link function combination was found. We therefore used a univariate approach, testing region, year and sex separately using a Kruskal–Wallis test for differences in medians with region or sex, and a Kendall rank correlation to test for trend over time. Because our univariate tests were unable to control for uneven sampling within the model, we tested for year differences within regions and tested for regional differences within years. Any groups with *N* < 6 were excluded (see Table 3).

### 2.4 Population structure and gene flow

Population differentiation was estimated between STGs in ARLEQUIN 3.5, using the pairwise F_ST_ estimator by Weir and Cockerham (1984) with the ‘number of different alleles (F_ST_‐like)’ option and 10,000 permutations and significance level set at 0.05. Analysis of Molecular Variance (AMOVA) was also performed in ARLEQUIN 3.5, with 10,000 permutations for significance and 1000 permutations for mantel test, using the ‘number of different alleles (F_ST_‐like)’ option for molecular distance.

We tested for isolation by distance and spatial autocorrelation using pairwise matrices of land distance and genetic distance estimates for each time point. Land distance was calculated using the GDISTANCE package in R to determine the least‐cost pathway between each pair of otters at each respective time point from a rasterised map of Great Britain with cell size set to 1 km^2^. On an irregularly shaped island like Great Britain, with multiple peninsulas separated by sea, land distance was deemed a more realistic measure of physical space between otters than Euclidean distance. Each land cell was given a resistance of 1 (sea cells were classified as ‘NoData’), such that the least‐cost resistance estimates calculated reflected the land distance between each pair of otters. For genetic distance, the proportion of shared alleles was calculated between pairs of otters using GenAlEx 6.5 (Peakall & Smouse, 2012). Mantel tests and Mantel correlograms were performed using the package VEGAN in R using Pearson's correlations and 10,000 permutations with an *α* = 0.05. The Holm correction for testing multiple *p*‐values was used for the Mantel correlograms, and breakpoints for distance classes were set to every 50 km from 0 to 800 km. Potential sex differences in isolation by distance and spatial autocorrelation were additionally evaluated by applying the same testing to subsets of the data including only adult females, and only adult males.

Gene flow between STGs was estimated using two different methods, for four time points spanning 1999–2014 (the first time point, 1994, was omitted due to a small sample size and lack of geographic coverage across the whole study region). Firstly, GENEPOP v4.6 (Rousset, 2008) was used to estimate the effective number of migrants (Nm), corrected for sample size, using the private alleles method developed by Barton and Slatkin (1986), which should be most sensitive to recent migration due to the rare nature of private alleles (Yamamichi & Innan, 2012). Nm was estimated across the whole dataset and pairwise between all STGs. These results will be referred to as nondirectional migration. Secondly, BayesAss v3.0 (Wilson & Rannala, 2003) was used to estimate pairwise directional gene flow between regions, allowing asymmetrical gene flow to be estimated for each pairwise comparison of populations. BayesAss uses a Bayesian algorithm to estimate recent migration (last 2–3 generations) between specified populations. Initially, the programme was run with the default values (of 0.1) for the three continuous parameters (migration rates (∆M), allele frequencies (∆A) and inbreeding coefficients (∆F)). Subsequently, these three parameters were adjusted until acceptance rates were within the recommended bounds of 20%–60% (Rannala, 2007), resulting in the selection of ∆M = 0.3, ∆A = 1.0 and ∆F = 1.0 for all time points. Three runs were performed per time point using different random seeds (starting points) with 10,000,000 MCMC iterations following a burn‐in of 1,000,000 MCMC iterations and a sample interval of 5000. Trace output files were recorded and used to monitor for mixing and convergence using TRACER v1.7.1 (Rambaut & Drummond, 2003) and the three runs were averaged to obtain the percentage of migrants between each region in a pairwise fashion. Migration rates between the Western Wales and Severn regions and the Northern and Eastern regions, respectively, were unlikely to provide reliable results as the pairwise F_ST_ between these regions was <0.05 and as such the results for these pairwise estimates were discarded (Faubet et al., 2007).

To determine the extent of population structure within UK otters we used two complementary approaches, one parametric and one nonparametric. The first was a Bayesian clustering algorithm implemented in STRUCTURE 2.3.4 (Pritchard et al., 2000), with no location prior, using the admixture model with correlated allele frequencies. All samples, regardless of collection year, were run together as one dataset for K = 1 to K = 13, with a burn‐in of 100,000 followed by 1,000,000 MCMC steps, running 10 replicates for each value of K. We chose K = 13 as the maximum K value based on the 11 river basin districts in Wales and England, plus Ireland and Scotland. The results were summarized using STRUCTURE HARVESTER v 0.6.94 (Earl & vonHoldt, 2012), and we used the method by Evanno et al. (2005) and the likelihood of K (Pritchard & Wen, 2003) to explore the most likely number of clusters present in the data. Individual admixture proportions were averaged across the 10 runs for each K using CLUMPAK using default parameters (Kopelman et al., 2015). Based on a cut‐off of 0.8 for the proportion of assignment to a specific cluster, we determined the number of ‘admixed’ individuals (Heppenheimer et al., 2018; Rutledge et al., 2010) at each time point for each value of K (i.e. any individual with less than 0.8 assignment to a single cluster was considered admixed). We used these data to quantify the percentage of admixed individuals across all clusters in the dataset at each time point. We note that a cut‐off at 0.8 is commonly used (e.g. Heppenheimer et al., 2018; Rutledge et al., 2010) but arbitrary. Other studies (e.g. Sanchez‐Donoso et al., 2014) have used even higher cut‐off values of 0.9, which would lead to the identification of a larger number of admixed individuals. However, a higher cut‐off comes at the cost of false positives, i.e. overestimation of admixture, due to inherent imprecision of the q value estimate from STRUCTURE, especially for moderate or low numbers of loci (Pritchard et al., 2000).

The second approach used was a Discriminant Analysis of Principal Components (DAPC), a multivariate approach (Jombart et al., 2009) that avoids making strong assumptions about the underlying genetic model (such as populations being in Hardy–Weinberg and linkage equilibrium). We used this approach as implemented in the R package *adegenet* (Jombart et al., 2010). Firstly, the number of de novo clusters was estimated using *find*.*clusters*, and for each model, a Bayesian Information Criterion (BIC) was computed. An optimal K or a range of K values was then selected based on the lowest BIC value or the steepness of the gradient between K's on the graph (Jombart et al., 2010; Miller et al., 2020). Subsequently, a DAPC was conducted using these predefined groups (K).

Progressive partitioning (Hobbs et al., 2011) based on Bayesian clustering results from STRUCTURE was conducted independently for the three time periods (2004, 2009 and 2014, i.e. excluding 1994 and 1999 for which samples were not available from the South West), based on data for all regions. As before, a burn‐in of 100,000 followed by 1,000,000 MCMC steps was used, restricting K to K = 2 for 5 replicate runs. For each run, individuals were assigned to one of the two clusters based on >50% assignment and each cluster went through another round of partitioning at K = 2 until the assignment of individuals was ~50% to each cluster.

## 3 RESULTS

### 3.1 Genetic variability by locus and River Basin district (RBD) region

Full genotypes (for 15 microsatellite loci) were obtained for all 193 samples newly analysed in this study (100% genotyping success rate), and re‐analysis of the 14 calibration samples allowed an additional 214 genotypes selected from previous studies (Hobbs et al., 2011; Stanton et al., 2014) to be adjusted such that the datasets could be merged and analysed as one. Across years, none of the loci showed significant evidence (*p* < 0.05) of null alleles at >2 of the 5 geographic regions, and therefore, all 15 loci were retained for further analysis. All 15 loci were polymorphic, with the number of alleles per locus ranging from 6 to 11, and observed heterozygosity per locus from 0.40 to 0.70 (Table 2).

**TABLE 2:** Genetic variability and information on loci.

Locus	Multiplex	Dye	N_A_	Size range (bp)	H_e_	H_o_
Lut435*	1	Fam	11	117–145	0.63	0.50
Lut453*	1	Hex	10	117–135	0.69	0.53
04OT05**	1	Hex	7	171–191	0.75	0.63
Lut717**	1	Ned	7	175–203	0.59	0.44
04OT22*	1	Fam	8	138–164	0.75	0.59
Lut604	2	Fam	7	127–137	0.72	0.62
Lut733	2	Fam	7	156–182	0.70	0.50
Lut615**	2	Fam	11	214–231	0.77	0.59
Lut902**	2	Hex	11	145–182	0.74	0.61
Lut782*	2	Ned	6	161–196	0.46	0.40
Lut818*	3	Fam	8	158–188	0.74	0.64
Lut701	3	Fam	9	193–248	0.66	0.49
Lut833*	3	Hex	7	154–176	0.75	0.70
Lut715	3	Hex	6	187–216	0.62	0.52
Lut832**	3	Ned	8	177–197	0.67	0.47
Mean	–	–	8.2	–	0.68	0.55

*Note*: Multiplex indicates which of the 3 multiplex mixes each locus belongs to, dye refers to the fluorescent dye used to label the PCR product, N_A_: the number of alleles detected at each locus, size range: states the size range of the alleles at that locus in number of base pairs (bp), H_e_: unbiased expected heterozygosity and H_o_: observed heterozygosity. Mean values across all loci for N_A_, H_e_ and H_o_ are given. A single asterisk* indicates that the locus was out of HWE in at least one RBD region; two asterisks** indicate that the locus was out of HWE in the majority (>3) of RBD regions.

**TABLE 3:** Genetic diversity statistics for all River Basin district regions (RBD regions) by year

RBD region	Year	*N*	*N* loci	H_e_	H_o_	N_A_	A_r_	pA_r_	A_r_ (*n* = 6)	pA_r_ (*n* = 6)	F_IS_
*Eastern*	*1994*	*1*	*15*	*0*.*53 ± 0*.*13*	*0*.*53 ± 0*.*13*	*1*.*53 ± 0*.*52*	*na*	*na*	*1*.*53*	*0*.*67*	*Na*
Eastern	1999	17	15	0.71 ± 0.03	0.69 ± 0.03	5.27 ± 1.53	5.22	1.12	4.23	1.00	0.027
Eastern	2004	7	15	0.72 ± 0.05	0.60 ± 0.05	4.67 ± 1.18	4.67	0.52	4.51	0.53	0.178
Eastern	2009	28	15	0.73 ± 0.03	0.65 ± 0.02	6.07 ± 1.39	5.41	0.85	4.43	0.78	0.107*w
Eastern	2014	21	15	0.71 ± 0.04	0.65 ± 0.03	5.47 ± 0.83	5.13	0.66	4.24	0.53	0.084
*Northern*	*1994*	*2*	*15*	*0*.*66 ± 0*.*04*	*0*.*70 ± 0*.*08*	*2*.*47 ± 0*.*52*	*na*	*na*	*2*.*47*	*0*.*36*	*Na*
Northern	1999	16	15	0.70 ± 0.02	0.62 ± 0.03	4.87 ± 1.19	4.87	0.58	3.97	0.68	0.110*w
Northern	2004	11	15	0.71 ± 0.02	0.65 ± 0.04	4.80 ± 1.08	4.34	0.42	4.16	0.44	0.079
Northern	2009	16	15	0.72 ± 0.02	0.64 ± 0.03	5.73 ± 1.83	5.49	0.79	4.43	0.71	0.115*w
Northern	2014	14	15	0.70 ± 0.02	0.67 ± 0.03	5.00 ± 0.85	5.00	0.42	4.08	0.38	0.045
Severn	1994	6	15	0.54 ± 0.04	0.49 ± 0.05	3.00 ± 0.53	3.00	0.47	3.00	0.11	0.109
Severn	1999	31	15	0.54 ± 0.03	0.48 ± 0.02	4.53 ± 0.99	3.93	0.02	3.10	0.08	0.112*w
Severn	2004	20	15	0.58 ± 0.03	0.51 ± 0.03	4.53 ± 0.64	3.50	0.12	3.35	0.12	0.137*w
Severn	2009	13	15	0.54 ± 0.04	0.51 ± 0.04	3.53 ± 0.92	3.53	0.08	3.01	0.07	0.054
Severn	2014	14	15	0.57 ± 0.03	0.45 ± 0.03	4.00 ± 1.07	4.00	0.16	3.28	0.15	0.223**i
South West	2004	23	15	0.55 ± 0.05	0.52 ± 0.03	4.00 ± 1.46	3.20	0.06	3.09	0.08	0.061
South West	2009	30	15	0.56 ± 0.05	0.49 ± 0.02	4.73 ± 1.16	4.09	0.06	3.32	0.15	0.124*i
South West	2014	24	15	0.60 ± 0.04	0.55 ± 0.03	5.13 ± 0.83	4.67	0.15	3.65	0.17	0.095*w
Western Wales	1994	19	15	0.48 ± 0.05	0.44 ± 0.03	3.27 ± 0.80	2.77	0.23	2.77	0.2	0.083
Western Wales	1999	34	15	0.54 ± 0.04	0.51 ± 0.02	4.00 ± 0.76	3.71	0.08	3.13	0.07	0.061
Western Wales	2004	15	15	0.52 ± 0.04	0.46 ± 0.03	3.60 ± 1.12	3.08	0.01	2.97	0.02	0.122
Western Wales	2009	17	15	0.56 ± 0.04	0.52 ± 0.03	3.93 ± 0.80	3.77	0.05	3.20	0.07	0.075
Western Wales	2014	17	15	0.55 ± 0.03	0.53 ± 0.03	3.73 ± 0.96	3.61	0.06	3.04	0.06	0.028

*Note*: *N*, number of individuals; *N* loci, number of loci retained for analysis; H_e_, Nei's unbiased expected heterozygosity; H_o_, observed heterozygosity; N_A_, average number of alleles per locus; A_r_, allelic richness; pA_r_, private allelic richness; F_IS_, coefficient of inbreeding (Weir & Cockerham, 1984). Asterisks indicate significant deviation from 0 after FDR correction (*p < 0.05, **p < 0.01); w indicates likely Wahlund effect whereas i indicates inbreeding (based on comparison with F_IS2_, see Table S3 for detail). A_r_ and pA_r_ are reported twice, once with rarefaction based on the smallest sample size at each time point (excluding sample sizes <5), and once using *n* = 6 (12 genes). Data in italics derive from small sample sizes and should hence be treated with caution, na's indicate that a particular statistic could not be calculated for that sample size. Underlined years indicate that the time point includes the year stated ±1 year, to increase the sample size. Samples from Ireland or Scotland are not included here due to a lack of temporal coverage.

Observed heterozygosity was consistently and significantly smaller than expected heterozygosity across all loci (paired two‐sample *t*_14_ = 11.219, *p* < 0.001; Table 2), potentially indicating population substructuring (Jin & Chakraborty, 1995). When the loci were tested for HWE across all subpopulations and time points, all loci departed significantly from HWE expectations. When analysing each RBD region independently, different loci were found to deviate significantly from HWE in different RBD regions: 11 of the 15 loci deviated significantly from HWE in at least one RBD region but only 5 of those loci deviated significantly in the majority (>3) of RBD regions. Observed heterozygosity was also consistently smaller than expected heterozygosity across all STGs (Table 3). Across all regions (using data from 2009 & 2014 only) many pairs of loci showed LD in one or more regions, but none had significant LD in all five. This difference in the results between the pooled, and the RBD‐based analysis indicates a Wahlund‐type geographic population structure in the dataset.

**FIGURE 2:**
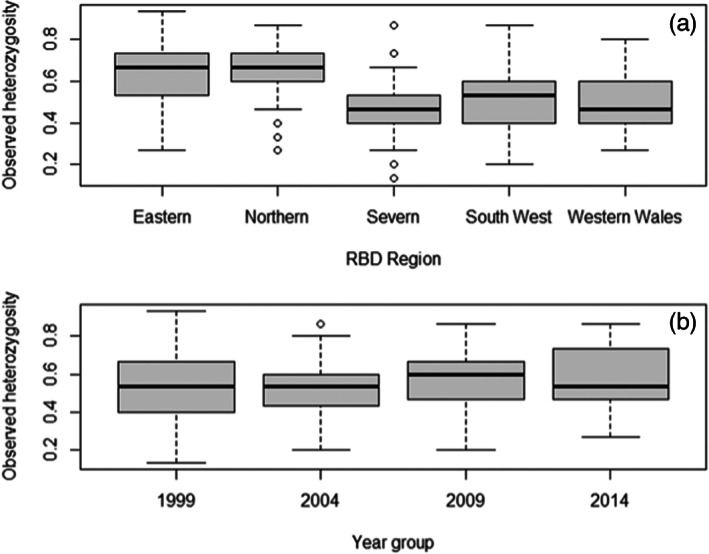
Boxplots showing variation in observed heterozygosity by RBD region and time. (a) Spatial variation by RBD region. Model results suggest significantly higher H_o_ in northern and eastern regions than in Severn South West and West Wales. Data shown exclude 1994 due to data deficiency across several regions; additionally, note that for South West region, there are no data for 1999. (b) Temporal variation by year (excluding 1994). Model results suggest no significant trend over time. In both plots, black lines indicate the median, grey boxes show the interquartile range, and whiskers show largest and smallest values excluding outliers, where outliers are defined as values that exceed 1.5 times the interquartile range and are shown as open circles.

All diversity indices suggested greater genetic diversity in the Eastern RBD regions (Eastern and Northern) than in the Western RBD regions (Severn, South West, Western Wales; Table 3). Genetic diversity within STGs as measured by H_e_ ranged from 0.48 (Western Wales, 1994) to 0.73 (Eastern, 2009) with an average of 0.61, while H_o_ ranged from 0.44 (Western Wales, 1994) to 0.70 (Northern, 1994; although the latter estimate was based only on two individuals and therefore should be treated with caution, see Table 3). The highest value for H_o_ from a region with a sample size >5 was 0.69 (Eastern region in 1999). Higher H_o_ was generally found in larger RBD regions (Northern and Eastern) while the lowest estimates were consistently found in the smallest RBD region (Western Wales; Kendall's Tau Correlation: Tau = 0.49, *p* = 0.004). This was not reflective of sample size (*N*; Kendall's Tau Correlation between H_o_ and *N*: Tau = −0.07, *p* = 0.648). GLM results for individual H_o_ (*F*_6,359_ = 15.63, *p* < 0.001) showed that RBD region explained a significant amount of variation in the data (*p* < 0.001), whereas year and sex were not significant (*p* > 0.05). Pairwise comparisons between regions showed significantly lower H_o_ in the Severn, South West and Western Wales regions when compared with Eastern and Northern regions (Tukey post hoc test: *p* < 0.001). There was no significant difference in H_o_ (Tukey test, *p* > 0.05) between the Eastern and Northern regions, or between the Severn, South West and Western Wales regions, indicating a significant West–East divide in this genetic diversity estimate (see Figure 2 for Region and Year comparison).

Other measures of diversity followed a similar spatial pattern, of higher genetic diversity in the Eastern than the Western RBD regions (Table 3). This was true for the average number of alleles per locus (N_A_), allelic richness (Ar) and private allelic richness (pAr). Ar and pAr were calculated using the smallest sample size across the regions for that time point as the resampling number and additionally calculated using the smallest (reasonable) sample size of any region at any time point (*N* = 6) as the resampling number, to allow comparisons unbiased by sample size. Although estimates were lower when the resampling size was smaller, as expected, there was a highly significant correlation between A_r_ and pA_r_ using both resampling techniques (Kendall's Tau = 0.78, *p* < 0.001), indicating that uneven sampling across time did not impact overall conclusions about regional genetic diversity. GLM results for A_r_ (*F*_5,13_ = 35.57, *p* < 0.001) showed the same pattern as those for H_o_, i.e. differences between geographic regions were highly significant, but temporal differences were not; subsequent pairwise comparisons between regions showed an identical pattern of significance and spatial differences as for H_o_, echoing the West–East divide in genetic diversity (see previous).

The F_IS_ estimates suggested significant inbreeding in the majority of RBD regions at certain time points, but many incidents of apparent inbreeding were likely due to the Wahlund effect (annotated with *i* and *w*, respectively, in Table 3, with additional detail in Table S3).

### 3.2 Population structure and gene flow

Tests of population differentiation showed that all STGs were significantly differentiated from each other apart from Severn‐West Wales in 2009 (Table 4). The degree of differentiation between RBD regions varied spatially. The significant pairwise F_ST_ values ranged from 0.24 (South West‐Western Wales, 2004) to 0.02 (Severn‐Western Wales, 1999 & 2004). All pairwise comparisons showed high F_ST_ values between RBD regions, apart from Eastern‐Northern and Severn‐Western Wales comparisons. By 2014, pairwise F_ST_ estimates remained high between the majority of RBD regions, with significant differentiation remaining between these subpopulations. Global estimates of F_ST_ at each time point estimated from AMOVA slightly decreased over time where all 5 RBD regions were included.

**TABLE 4:** Pairwise F_ST_ estimates (Weir & Cockerham, 1984) between RBD regions between 1999 and 2014.

Pairwise RBD regions	1999	2004	2009	2014
Eastern‐Northern	0.07	0.04	0.06	0.04
Eastern‐Severn	0.18	0.11	0.15	0.12
Eastern‐South West	n/a	0.09	0.13	0.11
Eastern‐Western Wales	0.16	0.14	0.15	0.13
Northern‐Severn	0.13	0.10	0.10	0.10
Northern‐South West	n/a	0.17	0.13	0.11
Northern‐Western Wales	0.13	0.12	0.11	0.11
Severn‐South West	n/a	0.19	0.19	0.18
Severn‐Western Wales	0.02	0.02	0.01^n.s.^	0.03
South West‐Western Wales	n/a	0.24	0.20	0.17
Global F_ST_	0.11	0.14	0.13	0.12
# RBD regions	4	5	5	5

*Note*: n/a: not enough data to estimate F_ST_ at that RBD‐year combination. All values were significant (*p* < 0.05) based on permutation tests of population differentiation in Arlequin (10,000 permutations), except where noted (n.s.). Bottom rows: global differentiation estimated from AMOVA, along with the number of RBD regions (# RBD regions) included in the analysis.

**FIGURE 3:**
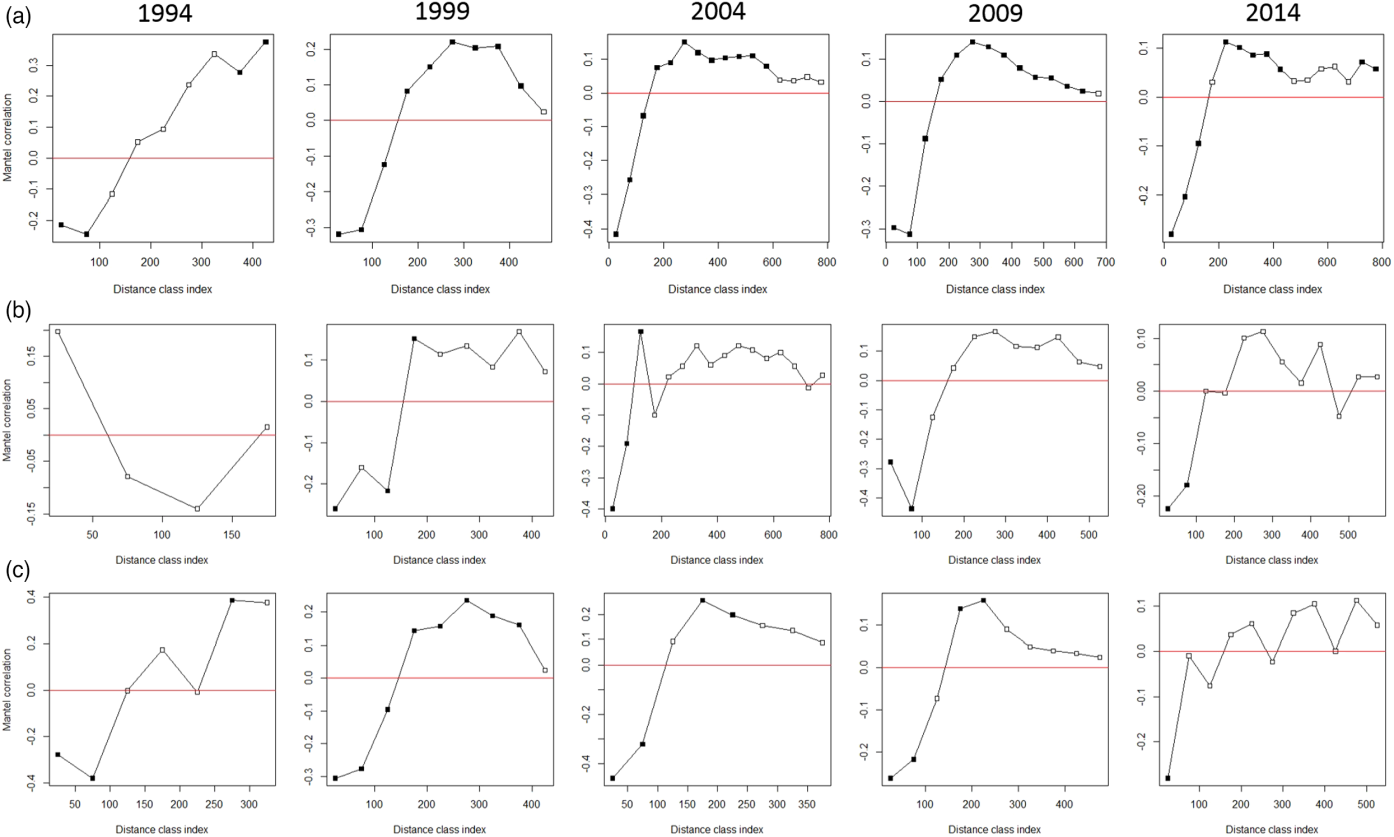
Mantel correlogram showing isolation by distance correlations across distance classes. (a) = all data. (b) = adult females only. (c) = adult males only. Black squares represent statistically significant spatial autocorrelation in that distance class, white squares represent nonsignificant results. Mantel correlation values >0 indicate positive spatial autocorrelation and mantel correlation values <0 show negative spatial autocorrelation.

Mantel tests for signals of isolation by distance (IBD) at each time point were not significant, indicating that there is no consistent overall IBD pattern across the UK. However, when we tested for spatial autocorrelation in the data using mantel correlograms it was possible to observe both negative and positive autocorrelation in different distance classes. Across all time points the relationship between Mantel's correlation and distance class was similar (Figure 3a) with negative spatial autocorrelation over the shorter distance classes (<150 km) and positive spatial autocorrelation at the larger distance classes (>150 km). Additionally, both adult females (Figure 3b) and adult males (Figure 3c) exhibited significant negative spatial autocorrelation over the first three distance classes (<150 km) at most time points, suggesting that dispersal is not sex‐biased but in fact undertaken by both sexes.

**FIGURE 4:**
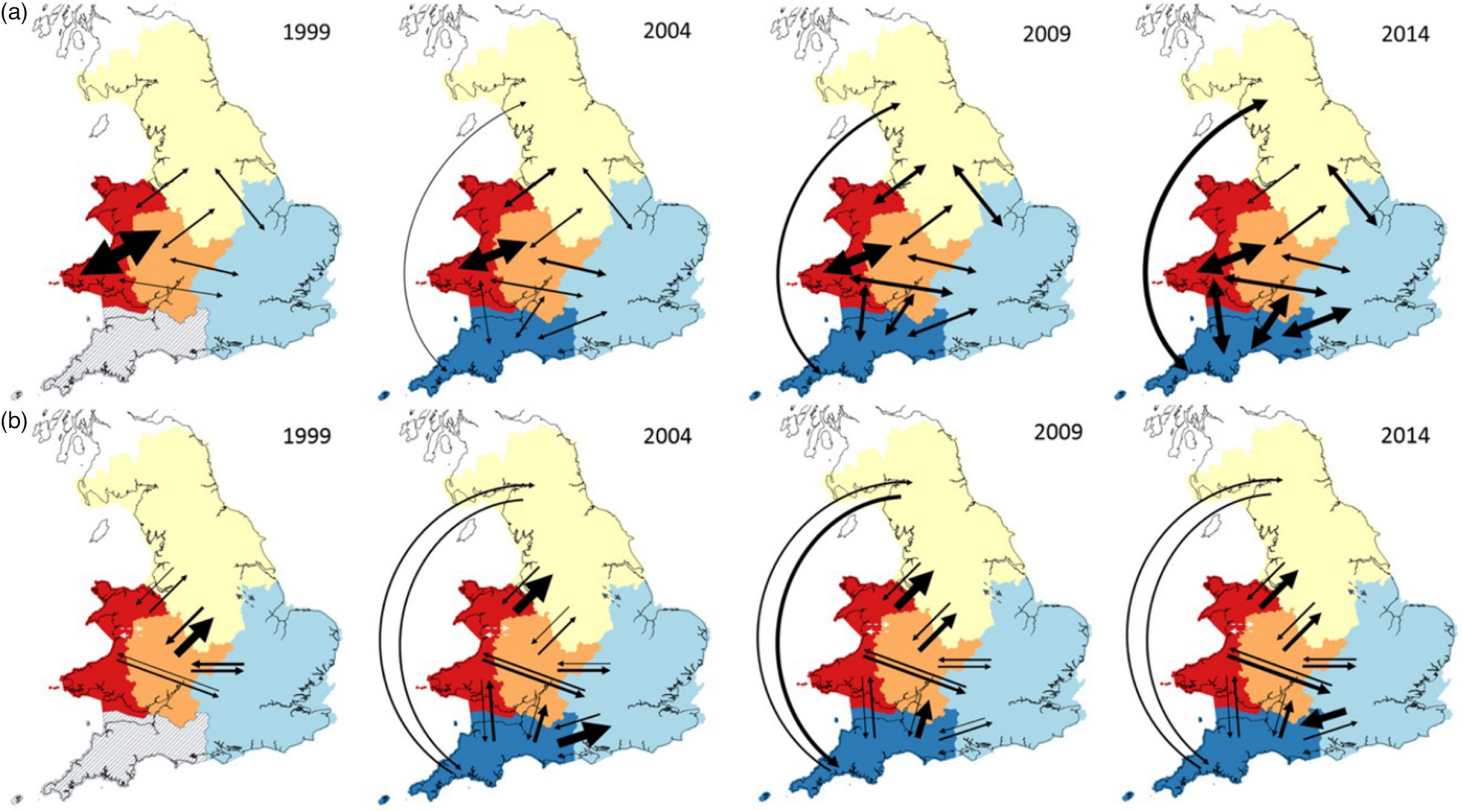
Gene flow between RBD regions at each of four time points (1999–2014). (a) Gene flow estimates from the private alleles method with arrows showing the migration between pairs of RBD regions and the arrow weight being directly proportional to the number of effectively migrating individuals. (b) Directional gene flow estimates from BayesAss with arrows showing the direction of effective migration and where arrow weight is directly proportional to percentage migration between populations. White and grey dashed arrows show regions where pairwise migration rates could not be reliably inferred due to low genetic differentiation. Regions are coloured as follows: Western Wales—red; Severn—orange; northern—yellow; eastern—light blue; South West—dark blue. Hatched lines indicate that a region was not included in the analysis at that time point.

Estimates of gene flow between RBD regions suggest an increase over time, whether using the nondirectional private alleles method (Barton & Slatkin, 1986; Figure 4a) or the Bayesian approach in BayesAss (directional migration; Figure 4b). The number of migrants estimated by the private alleles method approximately doubled at each time point between 2004 and 2014, indicating steadily increased gene flow over time (0.70, 0.73, 1.61 and 2.95 in 1999, 2004, 2009 and 2014, respectively). From the directional migration estimates from BayesAss, it is also possible to observe that in earlier time periods there was asymmetric gene flow with a higher effective migration rate from Wales and the South West into Eastern and Northern parts of England, than in the opposite direction. By the later time points, there was a more even exchange across the Western and Eastern regions. The estimates of gene flow from these two different approaches differ somewhat, likely due to their differing underlying methodologies combined with the fact that one is a uni‐directional estimate and the other a bi‐directional estimate. Nevertheless, both approaches revealed an increase in gene flow magnitude over time, suggesting that connectivity in UK otters increased over the study period.

Otters across the study area showed clear evidence of geographic substructuring. The value of K identified from the STRUCTURE runs using the Evanno ΔK method (Evanno et al., 2005) was K = 2. However, this method is biased towards K = 2 (Janes et al., 2017) and therefore other values of K were also explored (Figure 1; Figure S4) based on model likelihood, magnitude of ΔK and biological relevance (Cullingham et al., 2020), i.e. the known locations of the UK otter stronghold populations persisting through the 20th‐century declines (South West England, Wales, Southeast England, Northern England and Scotland; Hobbs et al., 2011; Stanton et al., 2014): the four values of K shown here all exhibit strong geographic clustering, with Wales and the South West being the most genetically distinct areas. Results from all explored values of K are shown in Figure S4. The percentage of individuals considered genetically admixed between clusters (Q < 0.8) increased over time (i.e. suggesting increased gene flow between areas in the UK) from 4% in 1994 (at K = 2, K = 3, K = 4a and K = 5b) to 16%, 17%, 24% and 23% in 2014 (at K = 2, K = 3 K = 4a and K = 5b, respectively). The alternate modes of K = 4 and K = 5 also showed increased admixture over time, albeit from a different starting point due to the partitioning of the Welsh cluster into two groups (for detail see Table S5). Correlation analysis for values at K = 3 suggests that increases in admixture were significant in Eastern, South West and Western Wales RBD regions (Kendall rank correlation: *p* = 0.029, 0.036, 0.029, respectively), whereas trends in Northern and Severn RBD regions were not significant (*p* > 0.05; for detail see Figure S6). Differences in admixture between RBD regions were only significant in 1999 (Kruskal Wallis chi‐square = 9.632, *p* = 0.025, when admixture was higher in Northern than in other regions); in all other years, admixture showed no difference between regions (*p* > 0.05). Differences in the degree of individual admixture between the sexes were also not significant (*p* > 0.05).

The number of genetic clusters in the data as inferred by Discriminant Analysis of Principal Components (DAPC) using the lowest BIC score as the model evaluation criterion was K = 8; however, these clusters were very difficult to rationalize biologically or spatially (data not shown) and thus we explored other values of K, using the gradient of the decline between BIC values as a guide. Comparison of the clusters inferred by DAPC (Figure S7) with those inferred from STRUCTURE (Figure 1) show close agreement for K = 2 and K = 3, and agreement with the major STRUCTURE modes for K = 4 and K = 5. This indicates that parametric and nonparametric methods agree for the strongest genetic divisions within the overall population (here strong agreement up to K = 3) but that assignments start to differ between methods as genetic divisions weaken (i.e. at higher values of K).

Progressive partitioning over selected time points where data for the whole study area was available (2004–2014) showed that the strength of regional differentiation differed with time (Figure S8). Despite this, the same four main subpopulations were identified in 2009 and 2014. In 2004, only three of the four main subpopulations were identified; however, sparse sampling in most of England at this time may have resulted in a lack of power to realize the Eastern‐Northern partition. The 2004 data also showed a significant partition between Cornwall and the rest of the South West (pairwise F_ST_ = 0.13, *p* < 0.001), more pronounced than at any subsequent time point. The major partitions exhibited significant genetic differentiation, as measured by pairwise F_ST_ across all three time points (*p* < 0.001 for all pairwise comparisons, for full data, see Table S9).

## 4 DISCUSSION

Our study represents comprehensive population genetic tracking of a recovering carnivore population over a twenty‐one‐year period. Quantifying changes in genetic structure over time allowed us to test theoretical predictions about demographic population recovery and ensuing genetic changes. Using the Eurasian otter as our case study, we predicted a weakening of genetic structure as anthropogenically fragmented populations reconnected as part of population recovery. Our use (predominantly) of roadkill samples may have biased our dataset toward younger males, which are assumed to disperse longer distances than other demographics (Philcox et al., 1999). This could lead to an overestimation of connectivity and diversity, but we found that the overarching population genetic structure showed surprisingly little change over time, with no increase in genetic variability (H_o_ and A_r_) within regions despite increased gene flow and admixture. These results suggest that the UK otter population is less functionally connected than previously presumed, perhaps due to landscape or other ecological/behavioural barriers impeding effective genetic mixing. Understanding such limitations in recovering populations is important with respect to managing and quantifying conservation successes.

### 4.1 Genetic variability

Spatial differences in both H_o_ and A_r_ were highly significant, with higher genetic variability in Eastern and Northern than in Western regions (Severn, South West and Western Wales) that persisted across the 21‐year study period.

In the absence of historic data predating the otter population crash, it is difficult to determine whether these spatial differences in diversity are historic or recent. However, translocations of otters into the Eastern and Northern regions are likely to have contributed to higher diversity in these regions. Population reinforcement projects have occurred in both regions, during which otters were released to bolster very small, residual populations that were deemed unlikely to recover without intervention. In the Northern region, 25 otters were released between 1990 and 1993; all were wild‐born otters rehabilitated following injury or abandonment and were translocated from widely dispersed UK locations to the Derwent and Esk catchments in Yorkshire (to the northeast of the Northern region; White et al., 2003). In the Eastern region, 117 otters were released between 1983 and 1999; all were captive bred in the UK, and most were released in East Anglia (to the east of the Eastern region), although later releases were also made on the upper Thames catchment (on the western edge of the Eastern region; Bonesi et al., 2013; Jefferies et al., 2000; Wayre, 1985). Although details of breeding stock are unknown, it appears likely that one of the utilized breeding lines may have included some Eurasian otters of non‐UK origin (Hájková et al., 2007). This could have led to the translocation of new alleles into Eastern and Northern regions and contributed to their higher genetic diversity (evidenced across all metrics), while their sparsely populated landscapes prior to reinforcement might also have facilitated natural immigration from neighbouring otter populations. No further population reinforcements have been made in the United Kingdom since the 1990s, and although a small number of rehabilitated individuals are released each year, these are typically kept within the area of origin (*pers*. *comm*. Grace Yoxon, Director of International Otter Survival Fund, UK). The persistence of higher genetic diversity in Eastern and Northern regions (despite the lack of any subsequent or ongoing translocations) suggests that the demographic connectivity implied by recent national otter surveys (Figure S1) has not resulted in significant mixing between these and adjacent regions.

Differences in the number of loci deviating from HWE and in LD—when calculated for the whole dataset versus by RBD region—are likely due to the Wahlund effect, i.e. an excess of homozygotes being observed due to sampling from a structured population, treating the whole dataset as one when subdivisions exist. A further important consequence of this Wahlund effect is allelic associations between loci across the total population, which can lead to signals of LD (Garnier‐Géré & Chikhi, 2013). If the signal reflected physical linkage between pairs of loci, then it would be expected to occur across all regions, whereas the patterns observed suggest other processes are occurring which emulate true physical linkage. One alternative explanation could be genetic admixture between regions. Admixture linkage disequilibrium has been described in unlinked loci due to differing allele frequencies in parental populations when there is gene flow between genetically distinct demes (Pfaff et al., 2001; Rybicki et al., 2002). Our two‐stage F_IS_ estimate added further clarity to this via establishing that many RBD regions are exhibiting Wahlund effects at specific time points. The F_IS_ values estimated for this dataset are higher than those previously estimated by Hobbs et al. (2011), suggesting either more subpopulation‐level inbreeding than previously thought or pronounced and sustained population structuring of UK otters (i.e. Wahlund effects). The latter is consistent with the relative lack of admixture of the subpopulations, which may be an indication that the UK otter population is demographically reconnected but still in an early genetic recovery phase, with individuals of differing genetic descent moving into RBD regions but an admixture of the genotypes yet to occur. Being able to disentangle inbreeding from the Wahlund effect is important in a conservation context and emphasizes the importance of understanding the population structure present, as well as the distribution of a species relative to the sampling area. Without this understanding, accidental sampling of individuals from genetically distinct demes as ‘one population’ even at low levels can have wide‐ranging effects on HWE, LD and F_IS_ estimates (Waples, 2015). Misinterpretation of significant results for any of the above could result in false assumption of inbreeding or natural selection within a population and may result in unnecessary interventions or designation of management units.

Several other studies across Europe have used similar methods to estimate the genetic diversity and population structure of otters at various spatial scales. In our study, across all samples, the average number of alleles (N_A_) was high compared with other European studies, whereas observed heterozygosity was relatively low (Lanszki et al., 2008; Mucci et al., 2010). This difference could be due to the significant substructuring and differentiation between regions in the UK, with the past population bottleneck and subsequent genetic drift leading to different suites of alleles being present in the different regions and resulting in a high N_A_ when looking at all the data as a whole. Consistent with this, diversity within the UK subpopulations is similar to that observed within population strongholds in the fragmented French otter population (Pigneur et al., 2019). The only other study in Europe with a N_A_ higher than ours is Arrendal et al. (2004), which similarly sampled across the whole of Sweden (and part of Norway), including populations that were highly genetically distinct from one another and that had received introductions. When split by spatial–temporal grouping (STG), N_A_ showed higher diversity in eastern regions than western ones within our study, but the overall range of values was within that for otter populations in countries across Europe (Mucci et al., 2010).

### 4.2 Population structure and gene flow

Both clustering analyses of population structure (STRUCTURE and DAPC) showed that otters in the United Kingdom have maintained a highly genetically structured population despite increased connectivity between subpopulations through range expansion. At higher values of K, the two methods deviated slightly in their spatial‐clustering pattern, which could be due to the assumptions made of Hardy–Weinberg equilibrium and/or linkage equilibrium in the Bayesian clustering model implemented in STRUCTURE, which does not exist in the DAPC model (Jombart et al., 2009). For both approaches, the inferred genetic clusters reflect known otter stronghold populations and agree with previous studies on the genetic structure of the UK otters (Hobbs et al., 2011; Stanton et al., 2014). This maintenance of population structure over time is also reflected in the predominantly high and temporally consistent pairwise F_ST_ estimates between most regions across all time points, in contrast to global F_ST_, which decreased over time. This could be due to the difference in the way that the two estimates are calculated (with pairwise F_ST_ using allele frequencies and our implemented AMOVA taking into account size distances among alleles; Excoffier et al., 1992) and also the larger sample size used in a global AMOVA. However, the global F_ST_ values only decrease marginally, and a global F_ST_ of 0.12 in 2014 still indicates a substantial level of inter‐regional differentiation. Strong spatial genetic structuring in the population therefore persists, despite an increase in gene flow over time (shown by all indicators), an increase in overall admixture rates at all values of K and significant increases in individual admixture rates in most regions.

There are few empirical studies that explicitly quantify changes in genetic structure over time during contemporary population expansions, and therefore, the body of work with which to compare our results is limited. However, the maintenance of genetic structuring seen in UK otters over more than 20 years is somewhat unexpected. For example, the rapid disintegration of genetic structuring was found in brown bears (*Ursus arctos*) in Finland by Hagen et al. (2015), over a similar time period. In that study, brown bears in Finland exhibited a rapid loss of population structure between 1996 and 2010 (15 years), as shown by decreasing pairwise F_ST_ values over time between the identified northern and southern genetic clusters. These changes to the genetic structure of the population were estimated to have occurred over only 1.5 generations. In our study, we observed much less pronounced changes to population structure over a similar sampling time period and a smaller study area (Finland is 338, 424 km^2^; Great Britain is 209,31 km^2^). Commonly used generation times in brown bears and otters are 11 years (Nilsson, 2013) and 3 years (Randi et al., 2003), respectively. Even based on the more conservative generation length estimates developed by Pacifici et al. (2013), the average generation length of the Eurasian otter is 7.6 years, whereas for the brown bear it is 16.4 years, indicating that despite the study lengths covering a similar time frame, in otters this should equate to a larger number of generations and therefore more opportunities for genetic mixing. In another study of brown bears, Schregel et al. (2017) found that high genetic differentiation persisted in Sweden and Norway between 2006 and 2013, despite the spatial connectivity of individuals across their study area. Consistent with our findings from UK otters, this illustrates that demographic connectivity is not always a reliable indicator of genetic connectivity in recovering populations of endangered species. Unlike UK otters and Fennoscandian brown bears, populations of many other endangered mammals have not yet reached spatial connectivity. Our findings are therefore relevant to the ongoing and future conservation of other recovering species such as grey wolves in southern and central Europe (Hindrikson et al., 2017) and central European lynx (Mueller et al., 2022).

There are several factors that could contribute to the long‐term maintenance of population structure in UK otters. Firstly, the landscape in the United Kingdom has changed significantly over the 40 years that otters were absent from large areas. Increased urbanization, human population size, number of roads, volume of traffic and alterations to rivers could all affect the realized connectivity between subpopulations. However, there is also a lag time for the genetic signatures left by old barriers to disappear and those created by new barriers to become detectable (Landguth et al., 2010). When assessing the effects of barriers to gene flow, the number of generations since (positive or negative) changes in barrier permeability need to be taken into account, as well as the dispersal ability of the species, as both of these factors have been shown to affect the rate at which such genetic signatures change (Landguth et al., 2010). It is assumed that otters can disperse over relatively large distances, as several tracking studies have recorded individuals moving tens of kilometres in one night (Green et al., 1984; Jenkins, 1980; Quaglietta et al., 2013), therefore dispersal limitation should be less likely in otters than in other less mobile species. Secondly, isolation in fragmented populations may have also affected other aspects of otter ecology through genetic drift—for example, Kean et al. (2017) found regionally differentiated scent odour profiles in the UK, which reflected genetic structure within the population. The discovery of these dialects is important given that otters communicate predominantly by scent material left in their spraint (Trowbridge, 1983). Scent differences apparently signalling age, reproductive status, sex and even individual identity have previously been described (Kean et al., 2011). These differing regional scent ‘dialects’ could be restricting gene flow, if otters are preferentially mating with otters of similar scent. Under this scenario, intrinsic aspects of otter behaviour may contribute to the maintenance of genetic structuring, despite the re‐establishment of spatial connectivity.

The results from our spatial autocorrelation analysis showed similar patterns at each time point (negative spatial autocorrelation in distance classes at small spatial scales, suggesting local dispersal). Given the territorial nature of adult otters (Erlinge, 1968), it would be expected that as a local population grows and reaches carrying capacity, individuals would need to move further to establish their own territory (Sjöåsen, 1997), leading to a gradual increase in the distance over which we observe negative spatial autocorrelation. Instead, our results showed no change over time, suggesting that even though populations are assumed to be approaching carrying capacity in some areas, dispersal distance has remained largely unchanged. A lack of density‐dependent dispersal in otters might explain this result but seems unlikely given that this phenomenon has been observed in over 70% of mammal species studied (reviewed in Matthysen, 2005). Furthermore, density‐dependent dispersal was also proposed as a mechanism for the short dispersal distances detected by Quaglietta et al. (2013) in otters in Portugal. An alternative explanation for our findings could be that improvements in river water and habitat quality have led to increased local carrying capacities, for instance through the recovery of prey populations (e.g. brown trout, Monteith et al., 2005). At higher prey densities, otter range size may decrease (Néill et al., 2009; Sidorovich, 1991), as has been observed for some other carnivore species such as the Eurasian lynx (Herfindal et al., 2005), countering increased dispersal distances associated with expanding populations. Our analysis of adult females and adult males suggests that both sexes disperse, as they exhibit similar patterns of negative spatial autocorrelation in the smaller distance classes (<150 km). These findings are contrary to previous evidence from radio tracking (which suggested male‐biased dispersal in Eurasian otters; Quaglietta et al., 2013) but are similar to the limit of gene flow detected for both sexes in Scotland by Dallas et al. (2002). We note, however, that our spatial autocorrelation analyses represent a relatively simplistic approach to explore spatial patterns of relatedness. Given that the landscape across Great Britain is highly heterogeneous, further analysis using a landscape genetics approach utilizing habitat‐specific resistance values should be undertaken to further elucidate otter dispersal in the UK.

## 5 CONCLUSIONS

Between surveys carried out in the late 1970s and 2010, the otter population in Great Britain expanded from small stronghold populations and near extinction in some areas (i.e. East Anglia) to an almost continuous distribution (Crawford, 2010; Strachan, 2015). This large‐scale population recovery was proclaimed a success for policy and practice, where changes in pollution control and the improvement of river and riparian habitats (Crawford, 2010) have supported a largely natural population expansion. Our study shows that this spatial connectivity has not translated into genetic connectivity in the manner or speed expected, with the population having retained the strong spatial genetic structuring observed earlier on in the recovery process (Hobbs et al., 2011; Stanton et al., 2014). The appearance of a spatially continuous population may therefore give a false sense of security with respect to genetic robustness, since in fact the population remains vulnerable, comprised of genetically fragmented subpopulations (Reed, 2004). Given the overall increasing levels of gene flow and admixture seen in this study, it may be that more time is all that is needed for the achieved spatial connectivity of the otter population to translate into genetic mixing. Future analysis of the data using landscape genetic techniques (Manel et al., 2003) or estimated effective migration surfaces (Petkova et al., 2016) may help identify any barriers to gene flow, whether extrinsic (e.g. landscape variables) or intrinsic (e.g. differences in scent profiles; Kean et al., 2017).

Demographic, behavioural and spatial barriers to the genetic mixing of subpopulations can lead to a time lag, where genetic recovery in terms of variability and connectivity requires much more time than a re‐occupation of range. Studies of spatial–temporal changes in genetic diversity and population structure during contemporary population expansions are still rare, perhaps due to the difficulties and costs of sampling over such large ranges, both spatially and temporally. However, such empirical studies are urgently needed to make predictions about population recovery progress and to set realistic goals for management and monitoring activities.

Our findings illustrate that spatial recovery of formerly endangered species may not necessarily imply that genetic recovery has occurred as well. Genetic recovery may require much longer than is apparent from spatial data alone leaving populations more vulnerable than they first appear. Newly published evidence from the 6th Otter Survey for Wales (Kean & Chadwick, 2021) has detected declines in otter signs across Wales, for the first time since surveys began. These new signals of potential population decline in Wales, alongside the lack of genetic recovery of otters across Wales and England illustrated by the current study, reinforce the value of population monitoring programmes that explicitly include genetic monitoring. Importantly, such studies are likely to provide a cornerstone for genetic monitoring programmes needed for the post‐2020 CBD biodiversity monitoring framework, wherein genetic diversity will be included for the first time (Hoban et al., 2021).

## CONFLICT OF INTEREST

The authors declare no conflict of interest.

## Supporting information


Appendix S1.

Appendix S2.

## Data Availability

A csv file of the raw genotype data with population information has been made available in the Supporting Information. See also the Table S2 for further details on all included samples. The same csv file is available on Dryad, see Thomas et al. 2022 in the reference list of this paper.
