# Supplementary material for: Country‐wide genetic monitoring over 21 years reveals lag in genetic recovery despite spatial connectivity in an expanding carnivore (Eurasian otter, Lutra lutra) population
Source: Evol Appl. 2022 Nov 15;15(12):2125–41. doi: 10.1111/eva.13505 (PMC9753835; doi:10.1111/eva.13505)
Supplement: Supplementary file 1 — Appendix S1. [file EVA-15-2125-s001.docx]

**Supplementary Information**

**Country-wide genetic monitoring over 21 years reveals lag in genetic recovery despite spatial connectivity in an expanding carnivore (Eurasian otter, *Lutra lutra*) population**

Nia E Thomas^1^, Frank Hailer^1,*^, Michael W Bruford^1^, Elizabeth A Chadwick^1^

^1^Organisms and Environment Research Division, School of Biosciences, Cardiff University, Sir Martin Evans Building, Museum Avenue, CF10 3AX Cardiff, Wales, UK

* Corresponding author: Frank Hailer, [HailerF@cardiff.ac.uk](mailto:HailerF@cardiff.ac.uk)

**Running title:** Population genetic recovery of UK otters

**Keywords:** Population recovery, microsatellites, population genetics, time lag, gene flow, genetic monitoring


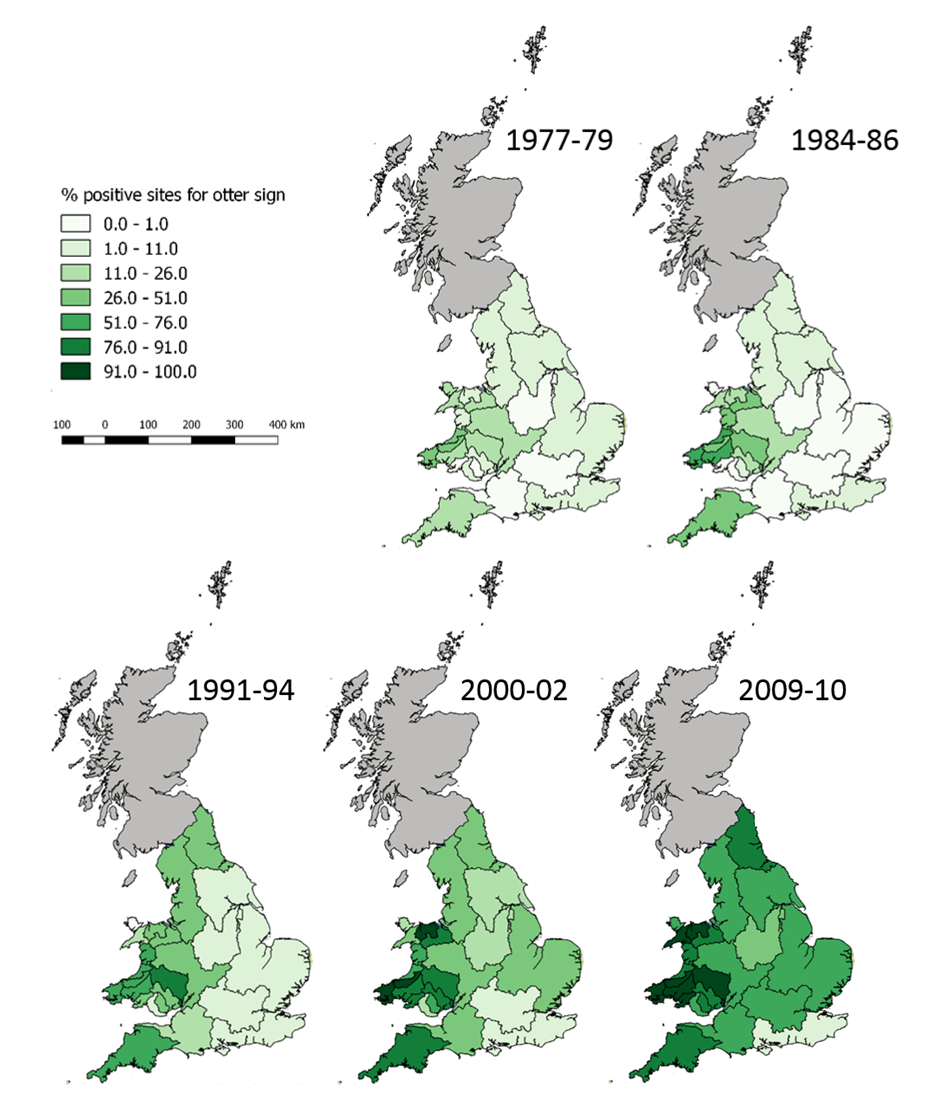


### **Suppl. Figure S1: National survey data for otters in Wales and England, based on data collated from surveys between 1977-2010.** Maps show increased frequency of otter sign, and increased distribution of otters over time. (Data for Wales from: Crawford et al. 1979, Andrews & Crawford 1986, Andrews et al. 1993, Jones & Jones 2004, Strachan 2015. Data for England from: Lenton et al. 1980, Strachan et al. 1990, Strachan & Jefferies 1996, Crawford 2003, 2010). The latest pop estimates for England and Wales are 2900 and 1000 respectively in 2010 (Mathews et al. 2018), up from 350 and 400 respectively in the mid-1980’s (Harris et al. 1995). These represent an over 8x increase for England and a 2.5x increase for Wales over 25 years. However, population estimates for otters in the UK are deemed to have very low reliability due to the methodology used in their calculation: % sites in each country recorded as being positive for otter sign at latest National Otter Survey multiplied by the length of river in that country (km) multiplied by 0.037 (otter density estimated in Green & Green 1987). The use of a single density estimate across such a large area of highly heterogenous landscape means that these estimates are seen as likely to be highly inaccurate (see Mathews et al. 2018 p 255-257 for further discussion on this).

### **Suppl. Table S2: Data allocation across the three studies**

| **Ind ID** | **Year** | **Study** | **RBD Region** |
| --- | --- | --- | --- |
| 7 | 1993 | Hobbs et al 2011 | Severn |
| 34 | 1993 | Hobbs et al 2011 | W Wales |
| 47 | 1993 | Hobbs et al 2011 | W Wales |
| 48 | 1993 | Hobbs et al 2011 | W Wales |
| 8 | 1994 | Hobbs et al 2011 | Severn |
| 11 | 1994 | Hobbs et al 2011 | W Wales |
| 21 | 1994 | Hobbs et al 2011 | W Wales |
| 25 | 1994 | Hobbs et al 2011 | W Wales |
| 26 | 1994 | Hobbs et al 2011 | W Wales |
| 31 | 1994 | Hobbs et al 2011 | Severn |
| 37 | 1994 | Hobbs et al 2011 | W Wales |
| 38 | 1994 | Hobbs et al 2011 | W Wales |
| 42 | 1994 | Hobbs et al 2011 | W Wales |
| 244 | 1994 | Hobbs et al 2011 | W Wales |
| 24 | 1995 | Hobbs et al 2011 | W Wales |
| 27 | 1995 | Hobbs et al 2011 | Severn |
| 29 | 1995 | Hobbs et al 2011 | Northern |
| 32 | 1995 | Hobbs et al 2011 | W Wales |
| 35 | 1995 | Hobbs et al 2011 | W Wales |
| 36 | 1995 | Hobbs et al 2011 | W Wales |
| 49 | 1995 | Hobbs et al 2011 | W Wales |
| 50 | 1995 | Hobbs et al 2011 | W Wales |
| 51 | 1995 | Hobbs et al 2011 | W Wales |
| 53 | 1995 | Hobbs et al 2011 | Eastern |
| 56 | 1995 | Hobbs et al 2011 | Severn |
| 60 | 1995 | Hobbs et al 2011 | W Wales |
| 246 | 1995 | Hobbs et al 2011 | Northern |
| 259 | 1995 | Hobbs et al 2011 | Severn |
| 143 | 1998 | Hobbs et al 2011 | W Wales |
| 147 | 1998 | Hobbs et al 2011 | W Wales |
| 148 | 1998 | Hobbs et al 2011 | W Wales |
| 149 | 1998 | Hobbs et al 2011 | W Wales |
| 151 | 1998 | Hobbs et al 2011 | W Wales |
| 152 | 1998 | Hobbs et al 2011 | Severn |
| 155 | 1998 | Hobbs et al 2011 | W Wales |
| 163 | 1998 | Hobbs et al 2011 | W Wales |
| 166 | 1998 | Hobbs et al 2011 | W Wales |
| 169 | 1998 | Hobbs et al 2011 | Severn |
| 174 | 1998 | Hobbs et al 2011 | Eastern |
| 176 | 1998 | Hobbs et al 2011 | Eastern |
| 177 | 1998 | Hobbs et al 2011 | Northern |
| 179 | 1998 | Hobbs et al 2011 | Northern |
| 184 | 1998 | Hobbs et al 2011 | Eastern |
| 185 | 1998 | Hobbs et al 2011 | W Wales |
| 187 | 1998 | Hobbs et al 2011 | Severn |
| 188 | 1998 | Hobbs et al 2011 | Severn |
| 190 | 1998 | Hobbs et al 2011 | W Wales |
| 191 | 1998 | Hobbs et al 2011 | W Wales |
| 192 | 1998 | Hobbs et al 2011 | Severn |
| 193 | 1998 | Hobbs et al 2011 | W Wales |
| 194 | 1998 | Hobbs et al 2011 | W Wales |
| 195 | 1998 | Hobbs et al 2011 | W Wales |
| 214 | 1998 | Hobbs et al 2011 | W Wales |
| 216 | 1998 | Hobbs et al 2011 | W Wales |
| 217 | 1998 | Hobbs et al 2011 | W Wales |
| 262 | 1998 | Hobbs et al 2011 | Northern |
| 264 | 1998 | Hobbs et al 2011 | Northern |
| 274 | 1998 | Hobbs et al 2011 | W Wales |
| 278 | 1998 | Hobbs et al 2011 | Northern |
| 281 | 1998 | Hobbs et al 2011 | Northern |
| 201 | 1999 | Hobbs et al 2011 | Eastern |
| 206 | 1999 | Hobbs et al 2011 | W Wales |
| 208 | 1999 | Hobbs et al 2011 | Severn |
| 210 | 1999 | Hobbs et al 2011 | W Wales |
| 211 | 1999 | Hobbs et al 2011 | W Wales |
| 215 | 1999 | Hobbs et al 2011 | W Wales |
| 218 | 1999 | Hobbs et al 2011 | W Wales |
| 225 | 1999 | Hobbs et al 2011 | Severn |
| 226 | 1999 | Hobbs et al 2011 | Severn |
| 227 | 1999 | Hobbs et al 2011 | Northern |
| 230 | 1999 | Hobbs et al 2011 | Eastern |
| 231 | 1999 | Hobbs et al 2011 | Eastern |
| 232 | 1999 | Hobbs et al 2011 | W Wales |
| 235 | 1999 | Hobbs et al 2011 | Northern |
| 238 | 1999 | Hobbs et al 2011 | Northern |
| 241 | 1999 | Hobbs et al 2011 | Severn |
| 242 | 1999 | Hobbs et al 2011 | W Wales |
| 243 | 1999 | Hobbs et al 2011 | W Wales |
| 248 | 1999 | Hobbs et al 2011 | Severn |
| 255 | 1999 | Hobbs et al 2011 | Eastern |
| 256 | 1999 | Hobbs et al 2011 | W Wales |
| 267 | 1999 | Hobbs et al 2011 | Other |
| 268 | 1999 | Hobbs et al 2011 | Northern |
| 386 | 1999 | Hobbs et al 2011 | Eastern |
| 254 | 2000 | Hobbs et al 2011 | Severn |
| 257 | 2000 | Hobbs et al 2011 | Eastern |
| 258 | 2000 | Hobbs et al 2011 | Severn |
| 260 | 2000 | Hobbs et al 2011 | Severn |
| 285 | 2000 | Hobbs et al 2011 | Northern |
| 286 | 2000 | Hobbs et al 2011 | Northern |
| 289 | 2000 | Hobbs et al 2011 | Severn |
| 290 | 2000 | Hobbs et al 2011 | Severn |
| 295 | 2000 | Hobbs et al 2011 | Severn |
| 299 | 2000 | Hobbs et al 2011 | Northern |
| 301 | 2000 | Hobbs et al 2011 | Severn |
| 304 | 2000 | Hobbs et al 2011 | Severn |
| 306 | 2000 | Hobbs et al 2011 | Northern |
| 307 | 2000 | Hobbs et al 2011 | W Wales |
| 314 | 2000 | Hobbs et al 2011 | Severn |
| 315 | 2000 | Hobbs et al 2011 | Severn |
| 317 | 2000 | Hobbs et al 2011 | Severn |
| 319 | 2000 | Hobbs et al 2011 | W Wales |
| 320 | 2000 | Hobbs et al 2011 | Severn |
| 321 | 2000 | Hobbs et al 2011 | Northern |
| 323 | 2000 | Hobbs et al 2011 | Eastern |
| 324 | 2000 | Hobbs et al 2011 | W Wales |
| 325 | 2000 | Hobbs et al 2011 | Eastern |
| 327 | 2000 | Hobbs et al 2011 | Severn |
| 328 | 2000 | Hobbs et al 2011 | Eastern |
| 331 | 2000 | Hobbs et al 2011 | Eastern |
| 333 | 2000 | Hobbs et al 2011 | W Wales |
| 335 | 2000 | Hobbs et al 2011 | Severn |
| 342 | 2000 | Hobbs et al 2011 | Severn |
| 346 | 2000 | Hobbs et al 2011 | Eastern |
| 350 | 2000 | Hobbs et al 2011 | Severn |
| 351 | 2000 | Hobbs et al 2011 | Severn |
| 354 | 2000 | Hobbs et al 2011 | Severn |
| 356 | 2000 | Hobbs et al 2011 | W Wales |
| 358 | 2000 | Hobbs et al 2011 | W Wales |
| 361 | 2000 | Hobbs et al 2011 | Eastern |
| 362 | 2000 | Hobbs et al 2011 | Severn |
| 366 | 2000 | Hobbs et al 2011 | W Wales |
| 367 | 2000 | Hobbs et al 2011 | Eastern |
| 376 | 2000 | Hobbs et al 2011 | Northern |
| 387 | 2000 | Hobbs et al 2011 | Eastern |
| 396 | 2000 | Hobbs et al 2011 | Other |
| 411 | 2000 | Hobbs et al 2011 | Severn |
| 412 | 2000 | Hobbs et al 2011 | Severn |
| 625 | 2004 | Hobbs et al 2011 | Severn |
| 626 | 2004 | Hobbs et al 2011 | Eastern |
| 627 | 2004 | Hobbs et al 2011 | Severn |
| 628 | 2004 | Hobbs et al 2011 | Northern |
| 630 | 2004 | Hobbs et al 2011 | W Wales |
| 632 | 2004 | Hobbs et al 2011 | W Wales |
| 634 | 2004 | Hobbs et al 2011 | W Wales |
| 635 | 2004 | Hobbs et al 2011 | Severn |
| 636 | 2004 | Hobbs et al 2011 | W Wales |
| 641 | 2004 | Hobbs et al 2011 | Severn |
| 642 | 2004 | Hobbs et al 2011 | Severn |
| 650 | 2004 | Hobbs et al 2011 | W Wales |
| 655 | 2004 | Hobbs et al 2011 | Northern |
| 656 | 2004 | Hobbs et al 2011 | Severn |
| 660 | 2004 | Hobbs et al 2011 | Severn |
| 661 | 2004 | Hobbs et al 2011 | Severn |
| 666 | 2004 | Hobbs et al 2011 | W Wales |
| 667 | 2004 | Hobbs et al 2011 | Severn |
| 669 | 2004 | Hobbs et al 2011 | Severn |
| 670 | 2004 | Hobbs et al 2011 | W Wales |
| 671 | 2004 | Hobbs et al 2011 | W Wales |
| 672 | 2004 | Hobbs et al 2011 | W Wales |
| 673 | 2004 | Hobbs et al 2011 | Severn |
| 676 | 2004 | Hobbs et al 2011 | Severn |
| 679 | 2004 | Hobbs et al 2011 | Severn |
| 680 | 2004 | Hobbs et al 2011 | Severn |
| 683 | 2004 | Hobbs et al 2011 | Northern |
| 684 | 2004 | Hobbs et al 2011 | Severn |
| 685 | 2004 | Hobbs et al 2011 | Severn |
| 686 | 2004 | Hobbs et al 2011 | Severn |
| 687 | 2004 | Hobbs et al 2011 | Northern |
| 688 | 2004 | Hobbs et al 2011 | Northern |
| 689 | 2004 | Hobbs et al 2011 | Northern |
| 691 | 2004 | Hobbs et al 2011 | W Wales |
| 702 | 2004 | Hobbs et al 2011 | Severn |
| 706 | 2004 | Hobbs et al 2011 | W Wales |
| 708 | 2004 | Hobbs et al 2011 | W Wales |
| 710 | 2004 | Hobbs et al 2011 | W Wales |
| 744 | 2004 | Hobbs et al 2011 | Northern |
| 745 | 2004 | Hobbs et al 2011 | Northern |
| 748 | 2004 | Hobbs et al 2011 | W Wales |
| 787 | 2004 | Hobbs et al 2011 | Northern |
| 856 | 2004 | Hobbs et al 2011 | Northern |
| 957 | 2004 | Hobbs et al 2011 | W Wales |
| 99634 | 2004 | Hobbs et al 2011 | South West |
| 99647 | 2004 | Hobbs et al 2011 | South West |
| 99650 | 2004 | Hobbs et al 2011 | Eastern |
| 99654 | 2004 | Hobbs et al 2011 | South West |
| 99658 | 2004 | Hobbs et al 2011 | South West |
| 99659 | 2004 | Hobbs et al 2011 | South West |
| 99667 | 2004 | Hobbs et al 2011 | South West |
| 99685 | 2004 | Hobbs et al 2011 | Eastern |
| 99686 | 2004 | Hobbs et al 2011 | South West |
| 99687 | 2004 | Hobbs et al 2011 | South West |
| 99692 | 2004 | Hobbs et al 2011 | Other |
| 99695 | 2004 | Hobbs et al 2011 | South West |
| 99701 | 2004 | Hobbs et al 2011 | South West |
| 99714 | 2004 | Hobbs et al 2011 | South West |
| 99722 | 2004 | Hobbs et al 2011 | Eastern |
| 99732 | 2004 | Hobbs et al 2011 | South West |
| 99739 | 2004 | Hobbs et al 2011 | South West |
| 99745 | 2004 | Hobbs et al 2011 | South West |
| 99766 | 2004 | Hobbs et al 2011 | South West |
| 99778 | 2004 | Hobbs et al 2011 | South West |
| 99795 | 2004 | Hobbs et al 2011 | South West |
| 99796 | 2004 | Hobbs et al 2011 | South West |
| 99800 | 2004 | Hobbs et al 2011 | South West |
| 99811 | 2004 | Hobbs et al 2011 | Northern |
| 99813 | 2004 | Hobbs et al 2011 | Eastern |
| 99815 | 2004 | Hobbs et al 2011 | Eastern |
| 99832 | 2004 | Hobbs et al 2011 | South West |
| 99840 | 2004 | Hobbs et al 2011 | Other |
| 99846 | 2004 | Hobbs et al 2011 | South West |
| 99858 | 2004 | Hobbs et al 2011 | Other |
| 99905 | 2004 | Hobbs et al 2011 | Severn |
| 99911 | 2004 | Hobbs et al 2011 | South West |
| 99945 | 2004 | Hobbs et al 2011 | South West |
| 991037 | 2004 | Hobbs et al 2011 | Severn |
| 991064 | 2004 | Hobbs et al 2011 | Eastern |
| 1264 | 2009 | Current Study | Eastern |
| 1270 | 2009 | Current Study | Northern |
| 1271 | 2009 | Current Study | Eastern |
| 1272 | 2009 | Current Study | Eastern |
| 1274 | 2009 | Current Study | Eastern |
| 1276 | 2009 | Stanton et al 2014 | Northern |
| 1281 | 2009 | Current Study | W Wales |
| 1282 | 2009 | Current Study | Eastern |
| 1283 | 2009 | Stanton et al 2014 | Northern |
| 1293 | 2009 | Current Study | South West |
| 1296 | 2009 | Current Study | South West |
| 1302 | 2009 | Stanton et al 2014 | Severn |
| 1305 | 2009 | Current Study | South West |
| 1306 | 2009 | Current Study | South West |
| 1309 | 2009 | Current Study | South West |
| 1310 | 2009 | Current Study | South West |
| 1313 | 2009 | Current Study | South West |
| 1315 | 2009 | Current Study | South West |
| 1317 | 2009 | Current Study | South West |
| 1318 | 2009 | Current Study | Eastern |
| 1319 | 2009 | Stanton et al 2014 | Northern |
| 1320 | 2009 | Stanton et al 2014 | Northern |
| 1321 | 2009 | Current Study | Severn |
| 1323 | 2009 | Current Study | Severn |
| 1329 | 2009 | Current Study | Severn |
| 1330 | 2009 | Current Study | Eastern |
| 1331 | 2009 | Current Study | Eastern |
| 1332 | 2009 | Current Study | Eastern |
| 1333 | 2009 | Current Study | Eastern |
| 1336 | 2009 | Current Study | Severn |
| 1346 | 2009 | Current Study | Eastern |
| 1353 | 2009 | Current Study | Eastern |
| 1354 | 2009 | Current Study | South West |
| 1355 | 2009 | Current Study | South West |
| 1356 | 2009 | Current Study | South West |
| 1357 | 2009 | Current Study | South West |
| 1358 | 2009 | Current Study | South West |
| 1362 | 2009 | Current Study | Eastern |
| 1363 | 2009 | Current Study | Northern |
| 1364 | 2009 | Current Study | Eastern |
| 1366 | 2009 | Current Study | Northern |
| 1367 | 2009 | Current Study | South West |
| 1374 | 2009 | Current Study | Eastern |
| 1376 | 2009 | Current Study | Eastern |
| 1377 | 2009 | Current Study | Eastern |
| 1378 | 2009 | Current Study | Eastern |
| 1384 | 2009 | Current Study | South West |
| 1386 | 2009 | Stanton et al 2014 | Northern |
| 1393 | 2009 | Current Study | Eastern |
| 1398 | 2009 | Stanton et al 2014 | W Wales |
| 1399 | 2009 | Current Study | South West |
| 1402 | 2009 | Current Study | South West |
| 1403 | 2009 | Current Study | South West |
| 1405 | 2009 | Current Study | Northern |
| 1412 | 2009 | Current Study | South West |
| 1418 | 2009 | Current Study | Northern |
| 1420 | 2009 | Current Study | South West |
| 1423 | 2009 | Current Study | Eastern |
| 1424 | 2009 | Current Study | Eastern |
| 1426 | 2009 | Current Study | Eastern |
| 1427 | 2009 | Current Study | Eastern |
| 1430 | 2009 | Current Study | Severn |
| 1437 | 2009 | Current Study | W Wales |
| 1440 | 2009 | Current Study | Severn |
| 1441 | 2009 | Current Study | Eastern |
| 1442 | 2009 | Current Study | South West |
| 1444 | 2009 | Current Study | W Wales |
| 1446 | 2009 | Current Study | W Wales |
| 1448 | 2009 | Current Study | W Wales |
| 1450 | 2009 | Current Study | W Wales |
| 1451 | 2009 | Current Study | W Wales |
| 1452 | 2009 | Current Study | W Wales |
| 1456 | 2009 | Current Study | W Wales |
| 1457 | 2009 | Current Study | W Wales |
| 1458 | 2009 | Current Study | W Wales |
| 1460 | 2009 | Current Study | W Wales |
| 1464 | 2009 | Current Study | Eastern |
| 1465 | 2009 | Current Study | South West |
| 1466 | 2009 | Current Study | South West |
| 1470 | 2009 | Current Study | South West |
| 1472 | 2009 | Current Study | South West |
| 1473 | 2009 | Current Study | Northern |
| 1474 | 2009 | Current Study | Northern |
| 1475 | 2009 | Current Study | South West |
| 1487 | 2009 | Current Study | Eastern |
| 1490 | 2009 | Current Study | South West |
| 1498 | 2009 | Current Study | Severn |
| 1500 | 2009 | Current Study | Severn |
| 1523 | 2009 | Current Study | Severn |
| 1531 | 2009 | Current Study | Northern |
| 1534 | 2009 | Current Study | Northern |
| 1535 | 2009 | Current Study | Northern |
| 1541 | 2009 | Current Study | W Wales |
| 1554 | 2009 | Current Study | South West |
| 1563 | 2009 | Current Study | Eastern |
| 1564 | 2009 | Current Study | Eastern |
| 1567 | 2009 | Current Study | W Wales |
| 1568 | 2009 | Current Study | W Wales |
| 1669 | 2009 | Current Study | South West |
| 1700 | 2009 | Current Study | Northern |
| 1735 | 2009 | Current Study | W Wales |
| 1873 | 2009 | Current Study | Severn |
| 1991 | 2009 | Current Study | Severn |
| 2066 | 2009 | Current Study | Severn |
| 2277 | 2014 | Current Study | W Wales |
| 2278 | 2014 | Current Study | Severn |
| 2292 | 2014 | Current Study | Eastern |
| 2294 | 2014 | Current Study | Severn |
| 2310 | 2014 | Current Study | Northern |
| 2311 | 2014 | Current Study | Severn |
| 2314 | 2014 | Current Study | Northern |
| 2319 | 2014 | Current Study | Eastern |
| 2321 | 2014 | Current Study | W Wales |
| 2323 | 2014 | Current Study | W Wales |
| 2325 | 2014 | Current Study | Eastern |
| 2330 | 2014 | Current Study | South West |
| 2331 | 2014 | Current Study | Eastern |
| 2332 | 2014 | Current Study | Eastern |
| 2336 | 2014 | Current Study | South West |
| 2340 | 2014 | Current Study | South West |
| 2341 | 2014 | Current Study | South West |
| 2342 | 2014 | Current Study | South West |
| 2344 | 2014 | Current Study | Severn |
| 2345 | 2014 | Current Study | Severn |
| 2347 | 2014 | Current Study | Severn |
| 2348 | 2014 | Current Study | Severn |
| 2351 | 2014 | Current Study | Eastern |
| 2355 | 2014 | Current Study | South West |
| 2356 | 2014 | Current Study | South West |
| 2358 | 2014 | Current Study | South West |
| 2362 | 2014 | Current Study | Northern |
| 2365 | 2014 | Current Study | W Wales |
| 2370 | 2014 | Current Study | Severn |
| 2377 | 2014 | Current Study | South West |
| 2380 | 2014 | Current Study | Eastern |
| 2383 | 2014 | Current Study | Eastern |
| 2386 | 2014 | Current Study | W Wales |
| 2388 | 2014 | Current Study | W Wales |
| 2391 | 2014 | Current Study | W Wales |
| 2398 | 2014 | Current Study | South West |
| 2401 | 2014 | Current Study | South West |
| 2406 | 2014 | Current Study | Northern |
| 2407 | 2014 | Current Study | Northern |
| 2409 | 2014 | Current Study | Northern |
| 2411 | 2014 | Current Study | South West |
| 2416 | 2014 | Current Study | Northern |
| 2420 | 2014 | Current Study | South West |
| 2422 | 2014 | Current Study | Other |
| 2424 | 2014 | Current Study | Other |
| 2425 | 2014 | Current Study | Other |
| 2434 | 2014 | Current Study | Eastern |
| 2435 | 2014 | Current Study | Eastern |
| 2438 | 2014 | Current Study | Northern |
| 2440 | 2014 | Current Study | Eastern |
| 2443 | 2014 | Current Study | Eastern |
| 2449 | 2014 | Current Study | Severn |
| 2453 | 2014 | Current Study | Severn |
| 2455 | 2014 | Current Study | W Wales |
| 2459 | 2014 | Current Study | Northern |
| 2460 | 2014 | Current Study | Severn |
| 2462 | 2014 | Current Study | Eastern |
| 2463 | 2014 | Current Study | Eastern |
| 2465 | 2014 | Current Study | Eastern |
| 2466 | 2014 | Current Study | Northern |
| 2467 | 2014 | Current Study | Eastern |
| 2468 | 2014 | Current Study | Eastern |
| 2471 | 2014 | Current Study | Eastern |
| 2474 | 2014 | Current Study | W Wales |
| 2477 | 2014 | Current Study | W Wales |
| 2478 | 2014 | Current Study | W Wales |
| 2479 | 2014 | Current Study | W Wales |
| 2480 | 2014 | Current Study | Northern |
| 2481 | 2014 | Current Study | Northern |
| 2482 | 2014 | Current Study | Northern |
| 2483 | 2014 | Current Study | South West |
| 2484 | 2014 | Current Study | South West |
| 2485 | 2014 | Current Study | South West |
| 2490 | 2014 | Current Study | South West |
| 2493 | 2014 | Current Study | Eastern |
| 2503 | 2014 | Current Study | W Wales |
| 2504 | 2014 | Current Study | W Wales |
| 2508 | 2014 | Current Study | W Wales |
| 2515 | 2014 | Current Study | Severn |
| 2519 | 2014 | Current Study | W Wales |
| 2521 | 2014 | Current Study | W Wales |
| 2527 | 2014 | Current Study | Eastern |
| 2529 | 2014 | Current Study | South West |
| 2533 | 2014 | Current Study | Eastern |
| 2544 | 2014 | Current Study | South West |
| 2545 | 2014 | Current Study | South West |
| 2547 | 2014 | Current Study | Other |
| 2548 | 2014 | Current Study | Other |
| 2560 | 2014 | Current Study | Other |
| 2585 | 2014 | Current Study | Northern |
| 2593 | 2014 | Current Study | South West |
| 2594 | 2014 | Current Study | South West |
| 2600 | 2014 | Current Study | South West |
| 2605 | 2014 | Current Study | South West |
| 2620 | 2014 | Current Study | Severn |
| 2640 | 2014 | Current Study | Severn |

### **Suppl. Table S3:** F_IS_ values for each region at each time point with information on genetic assignment of the individuals within a region to major and minor geographic clusters. N = number of samples, Major geographic cluster = dominant genetic cluster for that region, Minor cluster = dominant genetic cluster for another region, % All = percentage of all individuals within a region assigned to that cluster (Q<0.5), % Non-admixed = percentage individuals assigned with Q>0.8 to that cluster. % Admixed = percentage of individuals assigned with Q<0.8 to that cluster. F_IS_ is the F_IS_ estimate including all individuals in the RBD region at that time point, F_IS_2 is the F_IS_ estimate using only the individuals genetically assigned to the main geographic cluster for that region, Reason is a putative explanation for the significant F_IS_ values, taking into account any differences between F_IS_ and F_IS_2. Asterisks denote significance level after FDR correction with *<0.05 and **<0.01.

|  |  |  | Major | Geographic | Cluster | Minor | Cluster |  |  |  |
| --- | --- | --- | --- | --- | --- | --- | --- | --- | --- | --- |
| Region | Year | N | % All | % non-admixed | % Admixed | % All | % non-admixed | F_IS_ | F_IS_2 | Putative explanation |
| Eastern | 1999 | 17 | 100% | 88% | 12% | 0% | 0% | 0.027 | 0.027 | - |
|  | 2004 | 7 | 71% | 71% | 0% | 29% | 29% | 0.178 | 0.105 | - |
|  | 2009 | 28 | 93% | 86% | 7% | 7% | 4% | 0.107* | 0.083 | Wahlund |
|  | 2014 | 21 | 95% | 86% | 10% | 5% | 0% | 0.084 | 0.077 | - |
| Northern | 1999 | 16 | 88% | 75% | 13% | 13% | 6% | 0.11* | 0.093 | Wahlund |
|  | 2004 | 11 | 82% | 82% | 0% | 18% | 0% | 0.079 | 0.09 | - |
|  | 2009 | 16 | 94% | 56% | 38% | 6% | 6% | 0.115* | 0.108 | Wahlund |
|  | 2014 | 14 | 86% | 79% | 7% | 14% | 14% | 0.045 | -0.004 | - |
| Severn | 1994 | 6 | 100% | 100% | 0% | 0% | 0% | 0.109 | 0.109 | - |
|  | 1999 | 31 | 90% | 87% | 3% | 10% | 6% | 0.112* | 0.111 | Wahlund |
|  | 2004 | 20 | 85% | 80% | 5% | 15% | 15% | 0.137* | 0.15 | Wahlund |
|  | 2009 | 13 | 85% | 77% | 8% | 15% | 8% | 0.054 | -0.06 | - |
|  | 2014 | 14 | 93% | 86% | 7% | 7% | 0% | 0.223** | 0.241** | Inbreeding |
| South | 2004 | 23 | 96% | 91% | 4% | 4% | 0% | 0.061 | 0.043 | - |
| West | 2009 | 30 | 90% | 77% | 13% | 10% | 3% | 0.124** | 0.133* | Inbreeding |
|  | 2014 | 24 | 75% | 58% | 17% | 25% | 13% | 0.095* | 0.037 | Wahlund |
| Western | 1994 | 19 | 100% | 100% | 0% | 0% | 0% | 0.083 | 0.083 | - |
| Wales | 1999 | 34 | 100% | 91% | 9% | 0% | 0% | 0.061 | 0.061 | - |
|  | 2004 | 15 | 93% | 93% | 0% | 7% | 0% | 0.122 | 0.096 | - |
|  | 2009 | 17 | 100% | 88% | 12% | 0% | 0% | 0.075 | 0.075 | - |
|  | 2014 | 17 | 100% | 94% | 6% | 0% | 0% | 0.028 | 0.028 | - |


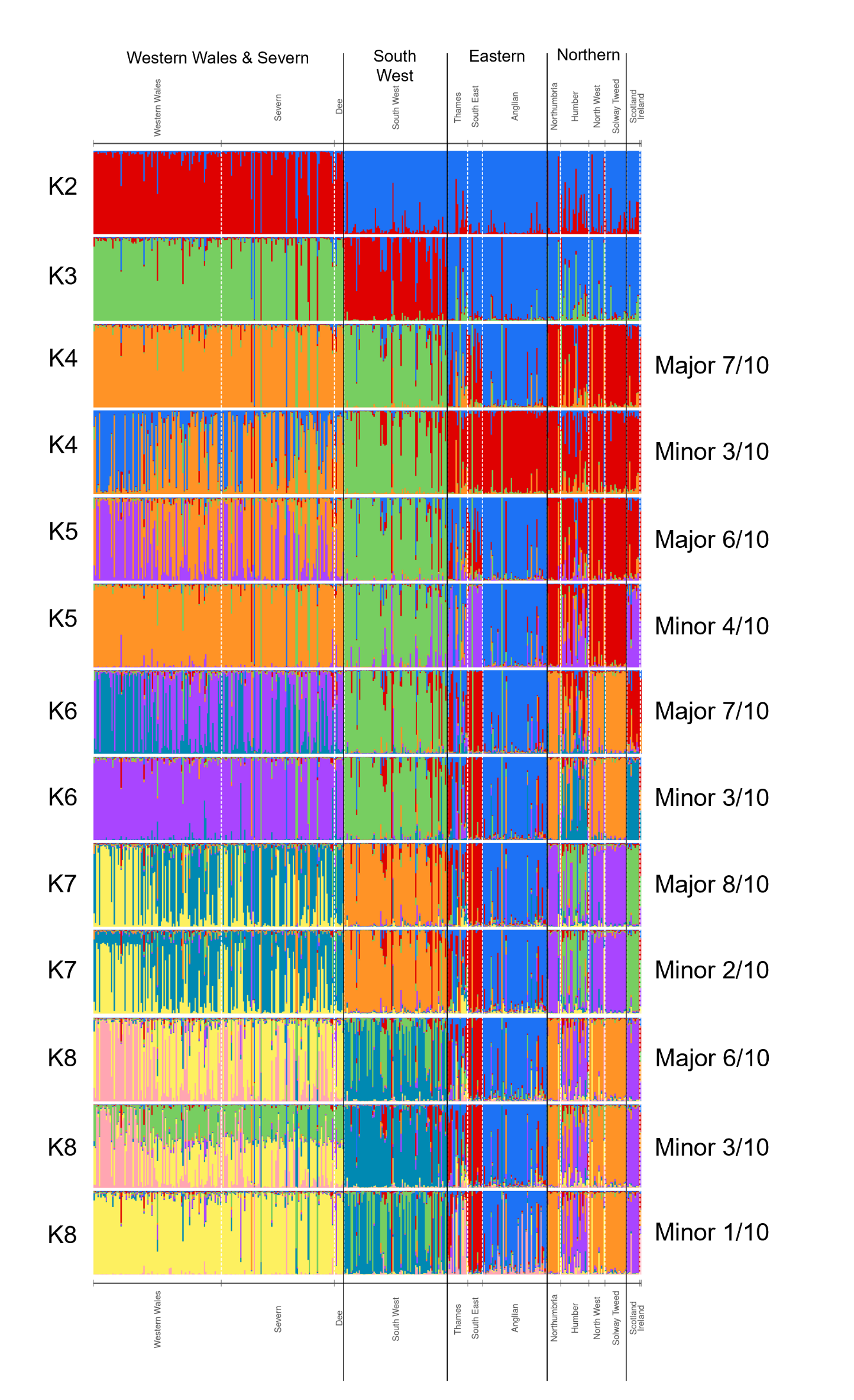


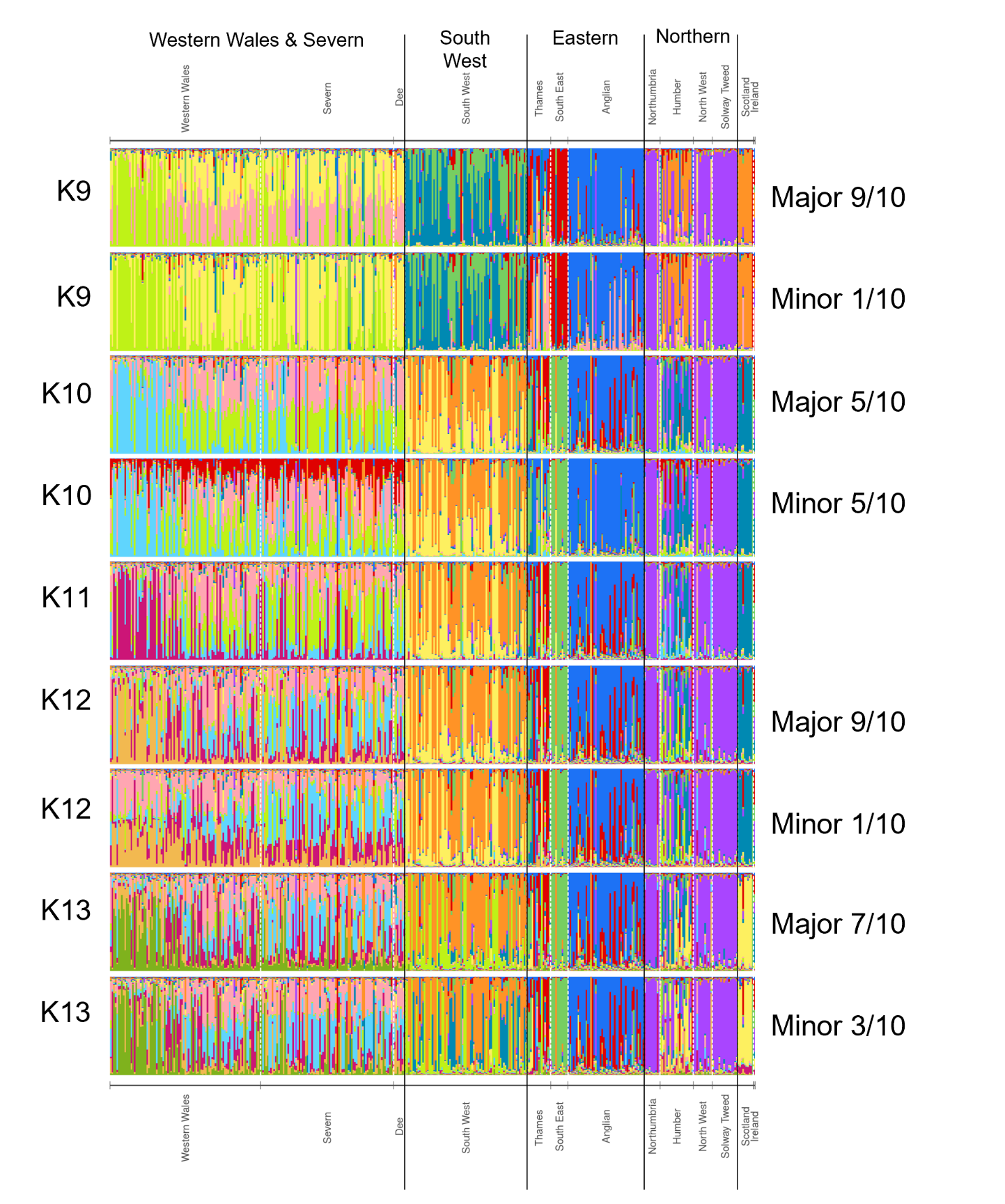


### **Suppl. Figure S4: STRUCTURE results across 10 repeats for each value of K (for K=2-13).**

Bar charts show individual assignments of each otter to the number of groups in a given run. The value of K is shown to the left of each bar chart, the number of runs attributed to that bar chart and whether this constitutes a major or a minor mode (determined by CLUMPAK) is shown on the right. Dashed white lines delineate the different river basin districts (RBDs) and solid black lines show larger regional groupings of RBDs.

### **Suppl. Table S5: Degree of admixture between genetic clusters at different values of K over time.** Percentages show the number of individuals considered to be genetically admixed between clusters at a given value of K. Both CLUMPAK modes for K=4 and K=5 (major and minor modes) are shown with notation matching the matching that in Figure 4. Genetically admixed was defined as an individual being <80% assigned to a single genetic cluster. 1994 and 1999 also include samples from the years directly adjacent (i.e. 1994 = 1993, 1994 and 1995) due to low sample sizes. Note that although not excluded, there was low N from Anglian and Northern regions in 1994.

|  |  |  |  | | | | | |
| --- | --- | --- | --- | --- | --- | --- | --- | --- |
| **Year** | **N** | **Area(s) excluded** | K=2 | K=3 | K=4a | K=4b | K=5a | K=5b |
| 1994 | 28 | South West | 4% | 4% | 4% | 17% | 14% | 4% |
| 1999 | 100 | South West | 9% | 10% | 10% | 21% | 22% | 15% |
| 2004 | 79 | None | 10% | 9% | 13% | 20% | 23% | 15% |
| 2009 | 104 | None | 14% | 17% | 18% | 28% | 32% | 21% |
| 2014 | 96 | None | 16% | 17% | 24% | 33% | 40% | 23% |


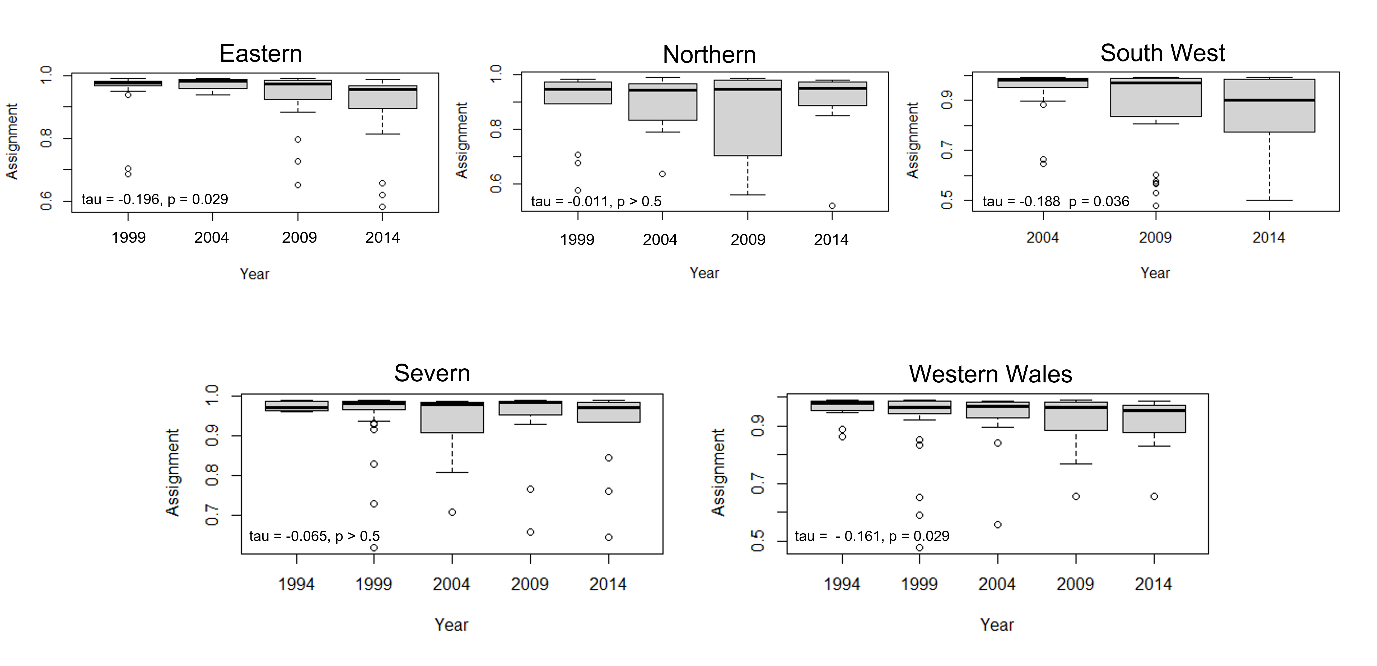


**Suppl. Figure S6: Change in individual admixture proportions between sampling year, for each RBD region.**

Individual plots are labelled by RBD region. Assignment is the admixture proportion for each individual, taken as the Q-value of the largest partition assignment per individual at K=3 (for bar charts in individual assignments see the STRUCTURE data in Suppl. Figure S4). Note that the y axis varies between plots, in order to maximise visualisation of change, and that year range differs between regions due to availability of data. Results of Kendall’s rank correlation analysis between assignment and year are indicated on each plot (tau, and p-value) and indicate significant trends in Eastern South West, and Western Wales.


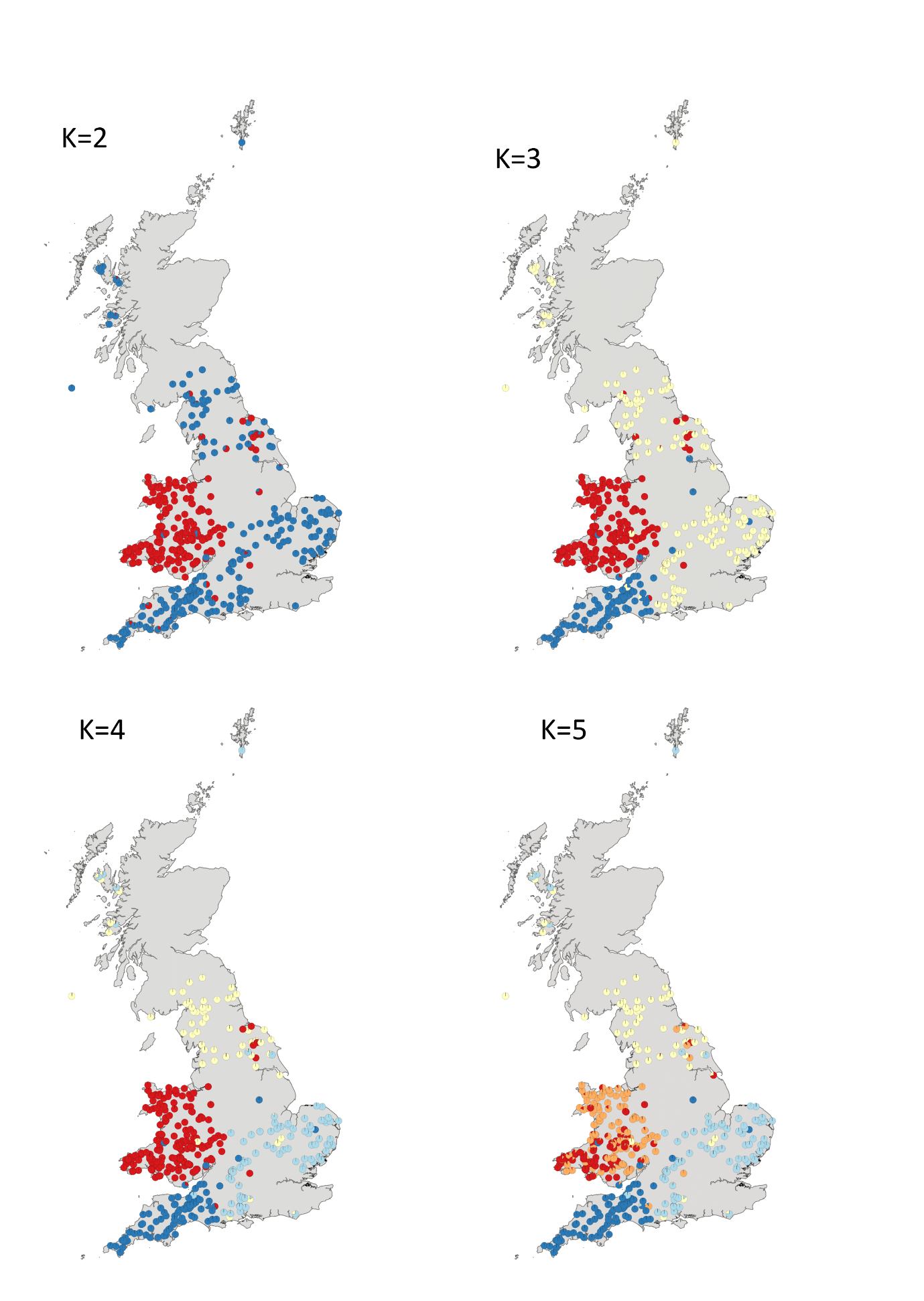


### **Suppl. Figure S7: Genetic clusters (K2-5) identified in UK otters sampled across 1999-2014 using Discriminant Analysis of Principal Components (DAPC).** Panels show results for K=2-5. Circles show the location of each genotyped otter, with colours indicating the estimated proportion of each genetic cluster.

###
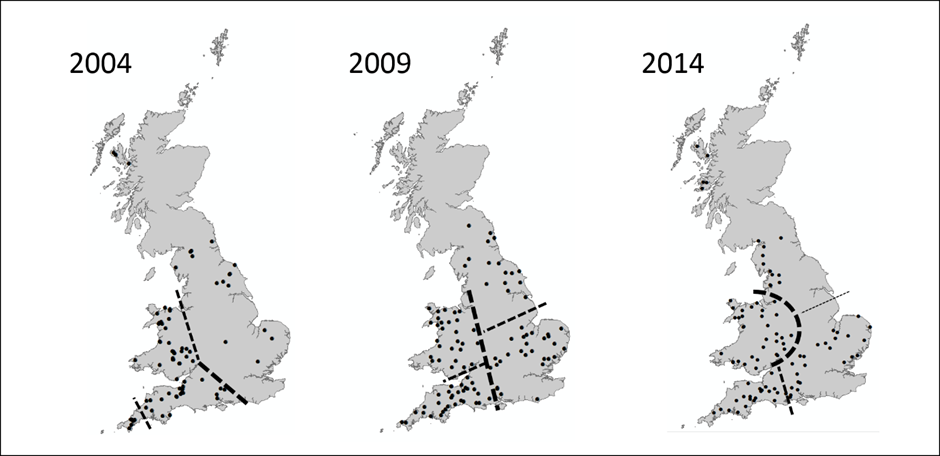
**Suppl. Figure S8: Progressive partitioning analysis of three time points where samples were available for the entire study area.**

*Main population partitions of UK otters as determined by progressive partitioning across three time points. Line thickness denotes the level of the partition with the thickest black line indicating the 1st partition in the data at each time point.*

### **Suppl. Table S9: Pairwise F_ST_ estimates (Weir & Cockerham 1984) between major partitions identified at each time point.** SW: Southwest England, Eng: Combined East and North England). ***: significantly differentiated at p<0.001.

| **2004** | **Wales** | **SW** | **Eng** |
| --- | --- | --- | --- |
| **Wales** | - | *** | *** |
| **SW** | 0.12 | - | *** |
| **Eng** | 0.16 | 0.24 | - |

| **2009** | **Wales** | **SW** | **Northern** | **Eastern** |
| --- | --- | --- | --- | --- |
| **Wales** | - | *** | *** | *** |
| **SW** | 0.23 | - | *** | *** |
| **Northern** | 0.13 | 0.13 | - | *** |
| **Eastern** | 0.22 | 0.20 | 0.07 | - |

| **2014** | **Wales** | **SW** | **Northern** | **Eastern** |
| --- | --- | --- | --- | --- |
| **Wales** | - | *** | *** | *** |
| **SW** | 0.21 | - | *** | *** |
| **Northern** | 0.12 | 0.12 | - | *** |
| **Eastern** | 0.14 | 0.14 | 0.06 | - |
